# AutoDock-GIST: Incorporating Thermodynamics of Active-Site Water into Scoring Function for Accurate Protein-Ligand Docking

**DOI:** 10.3390/molecules21111604

**Published:** 2016-11-23

**Authors:** Shota Uehara, Shigenori Tanaka

**Affiliations:** Department of Computational Science, Graduate School of System Informatics, Kobe University, 1-1 Rokkodai, Nada, Kobe, Hyogo 657-8501, Japan

**Keywords:** protein-ligand docking, virtual screening, grid inhomogeneous solvation theory (GIST), AutoDock4, binding-site water

## Abstract

Water plays a significant role in the binding process between protein and ligand. However, the thermodynamics of water molecules are often underestimated, or even ignored, in protein-ligand docking. Usually, the free energies of active-site water molecules are substantially different from those of waters in the bulk region. The binding of a ligand to a protein causes a displacement of these waters from an active site to bulk, and this displacement process substantially contributes to the free energy change of protein-ligand binding. The free energy of active-site water molecules can be calculated by grid inhomogeneous solvation theory (GIST), using molecular dynamics (MD) and the trajectory of a target protein and water molecules. Here, we show a case study of the combination of GIST and a docking program and discuss the effectiveness of the displacing gain of unfavorable water in protein-ligand docking. We combined the GIST-based desolvation function with the scoring function of AutoDock4, which is called AutoDock-GIST. The proposed scoring function was assessed employing 51 ligands of coagulation factor Xa (FXa), and results showed that both scoring accuracy and docking success rate were improved. We also evaluated virtual screening performance of AutoDock-GIST using FXa ligands in the directory of useful decoys-enhanced (DUD-E), thus finding that the displacing gain of unfavorable water is effective for a successful docking campaign.

## 1. Introduction

Water is an indispensable participant in the binding process of a protein and a small molecule [1,2,3,4,5,6,7,8]. In an in vivo environment, the active sites of a proteins are filled with water molecules, and thermodynamics of these water molecules are diverse and quite different from those of bulk water [9,10,11]. When a small molecule binds to a protein, it causes the displacement of water molecules from the active site to the bulk region, and the thermodynamics of this displacement process is a principal source of binding free energy of ligands [12,13,14]. For instance, a water molecule enclosed by hydrophobic residues of protein that cannot make appropriate hydrogen bonds is enthalpically unfavorable, and the displacement of such water earns an enthalpic contribution in binding free energy. On the other hand, a water molecule forming tight hydrogen bonds to hydrophilic residues of a protein is enthalpically favorable, and the displacement of such water may incur the penalty of protein-ligand binding. Thus, the role of active-site water molecules is widely appreciated in the study of molecular recognitions [15,16,17,18,19].

Computational approaches for analyzing active-site water properties have become essential to our better understanding of protein-ligand binding [20,21,22,23]. Many computational methods have been developed to predict the location of binding-site water and/or its binding properties [24,25,26,27,28,29]. In recent years, molecular dynamics (MD) based methods have led to important advances in the study of active-site water and its thermodynamic role in ligand binding. The early key contributions include WaterMap [30], STOW [31], WATsite [32], and other approaches [33,34]. These methods usually determine high-density water locations as a spherical site, termed the ‘hydration site’, by analyzing the MD trajectory of protein and explicit water molecules, and calculate various thermodynamic quantities. For example, WaterMap locates hydration sites using a clustering algorithm, and calculates enthalpic and entropic contributions of individual hydration sites based on inhomogeneous solvation theory (IST) [35,36]. The hydration site analysis (HSA) helps researchers intuitively understand crucial water upon ligand binding, although it cannot represent the complex shape of high-density water regions by a collection of spheres [37]. Moreover, there is another MD-based approach called grid inhomogeneous solvation theory (GIST) [37,38]. Instead of locating hydration sites, GIST discretizes the continuous distribution of water density and thermodynamic properties onto three-dimensional grids. Accordingly, compared to HSA based methods, GIST can capture the complex shape of water distribution, covering high- and low-occupancy water regions.

Protein-ligand docking simulation is a powerful tool for the rational and efficient design of small molecules in structure-based drug design (SBDD) [39,40,41]. The atom-atom pairwise potentials, used in most of the scoring functions of docking programs, give a relevant approximation of interaction energy between proteins and ligands. However, the accurate estimation of thermodynamics of water molecules is still challenging due to the highly expensive cost of computation for virtual screening [42]. In recent years, the precise modeling and scoring of water molecules has become a critical issue of protein-ligand docking [43,44,45]. For example, some early works introduced hydration water molecules which remain in the binding site and form hydrogen bonds to proteins and ligands into docking program, and improved docking performances [46,47,48]. However, thermodynamics of displaced water molecules are still underestimated or even ignored in protein-ligand docking. Many scoring functions of docking software, including AutoDock, use an implicit solvent model in the form of a continuous desolvation function [49,50,51] which cannot describe in homogeneousness of active-site water molecules. The thermodynamics of displaced water molecules is a fundamental component of protein-ligand binding that contributes not only to the binding affinity but also to the binding conformation of ligands, since the ligand replaces unfavorable water molecules more easily than tightly bound water molecules [52]. Thus, the appropriate description of active-site water molecules should be essential for the improvement of docking performance.

Here, we incorporate thermodynamics of active-site water molecules into AutoDock4 [53] by combining a new desolvation function based on grid inhomogeneous solvation theory (GIST), which is called AutoDock-GIST. The GIST-based desolvation function was designed to formulate the driving force for unfavorable water molecules displaced by the binding ligand. Similar desolvation functions were proposed in previous studies of WaterMap and GIST [30,38]. Notably, they estimated the affinity difference between the closely related congeneric pair of ligands, where the difference in binding affinity results from dominant contributions of solvation rather than protein-ligand interaction [30,38]. Following these two key studies, the present work attempted to estimate binding affinities of diverse ligands and to improve docking success rates by combining the scoring function of AutoDock and GIST-based desolvation function. Since AutoDock uses a gridded energy map for fast calculation of scoring function [54], the grid water properties of GIST are tractable to be incorporated into AutoDock. Furthermore, after calculating active-site water properties from single MD trajectory of the apoprotein and explicit water, the GIST-based desolvation function can be used for virtual screening campaign via docking with almost the same computational cost as in AutoDock4.

To validate the capability of our proposed scoring function, we study the complex system of coagulation factor Xa (FXa) and its small molecule inhibitors of 51 ligands which have experimentally measured binding affinities and X-ray crystal structures, including 28 ligands used in a previous work by WaterMap [30]. Using this dataset, we discuss the performance of AutoDock-GIST concerning the binding affinity estimation and the binding pose prediction. Furthermore, we evaluate the virtual screening performance, employing 793 active and 20,418 decoy compounds of FXa from the directory of useful decoys-enhanced (DUD-E) [55]. The results have revealed that scoring accuracy, docking success rate, and screening performance are significantly improved. Note that our work is a case study for a single target protein of FXa, but the finding generally supports the applicability of GIST for successful docking campaign.

## 2. Materials and Methods

### 2.1. Grid Inhomogeneous Solvation Theory (GIST)

**Figure 1:**
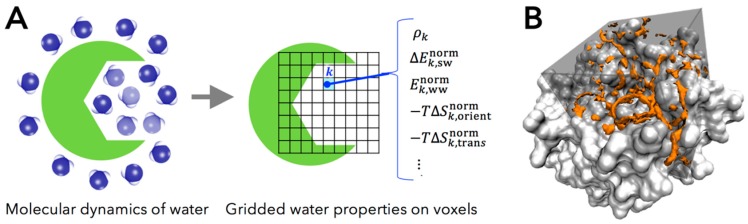
(**A**) Diagram of grid inhomogeneous solvation theory (GIST) calculation. The grid water properties of GIST are calculated using molecular dynamics (MD) trajectory of protein and explicit water; (**B**) The two-fold denser water regions (orange) than bulk in the active site of coagulation factor Xa (FXa) (gray) calculated by GIST. Figure prepared by using Visual Molecular Dynamics (VMD) [57].

Grid inhomogeneous solvation theory (GIST) is a powerful and tractable computational method to calculate the hydration structure and thermodynamics of water around macromolecules, proposed by Nguyen et al. [37]. The thermodynamic properties of water molecules can be calculated based on inhomogeneous solvation theory (IST) [35,36], using the snapshots of trajectories obtained from MD simulation of explicit water and protein. Most other computational methods, except GIST, use hydration site analysis (HSA) to identify the high-density and localized water region, called the hydration site. Although HSA-based approaches provide valuable insights into the role of specific water sites, they still have a significant limitation that they do not provide information on larger high-density water regions and other regions where the water density is low, rather than high, relative to bulk value [56]. To overcome these limitations, GIST discretizes IST onto a three-dimensional grid that fills the active site of protein, covering all occupancy regions of water (Figure 1). Thus, GIST provides more informative pictures of hydration water as the distribution of density and its thermodynamic properties.

GIST calculates various thermodynamic quantities of water molecules on the three-dimensional rectangular grid of cubic voxel k in the region of interest. The complete description of the GIST method is compiled in the original paper [37]. In the present work, we studied the following five properties of water molecules in voxel k, computed by GIST:

- $\rho_{k}$, the number density of oxygen atom of water molecule found in a voxel k, in units of the density in bulk region (i.e., the number density of bulk water $\rho_{\mathrm{bulk}}=1$).
- $\Delta E_{k,\mathrm{sw}}^{\mathrm{norm}}$, the mean energy of solute-water interaction per water molecule in a voxel k (kcal/mol/water). This quantity is referenced to bulk water, in the trivial sense that the energetic contribution of solute-water interaction is zero in bulk region.
- $E_{k,\mathrm{ww}}^{\mathrm{norm}}$, one-half the mean energy of water-water interaction per water molecule in a voxel k with all other water molecules (kcal/mol/water). The one-half factor prevents double counting of two water-water interactions and preserves the total energy of neat water being written as the sum of the single water energy [38].
$-T\Delta S_{k,\mathrm{orient}}^{\mathrm{norm}}$, first-order orientational entropy per water molecule in a voxel k (kcal/mole/water), referenced to bulk water (i.e., the orientational entropy of bulk water is set to be zero).
$-T\Delta S_{k,\mathrm{trans}}^{\mathrm{norm}}$, first-order translational entropy per water molecules in a voxel k (kcal/mol/water), referenced to bulk water (i.e., the translational entropy of bulk water is set to be zero).

Based on these quantities, thermodynamic properties of water molecules are described by the following equations. Here, we regard the interaction energy as an enthalpic contribution in this paper. The total enthalpy of a water molecule in a voxel k, relative to bulk, is defined as:

(1) $$\Delta H_{k}^{\mathrm{norm}}=\Delta E_{k,\mathrm{sw}}^{\mathrm{norm}}+2\left(E_{k,\mathrm{ww}}^{\mathrm{norm}}-E_{\mathrm{bulk},\mathrm{ww}}^{\mathrm{norm}}\right)\;$$

where $E_{\mathrm{bulk},\mathrm{ww}}^{\mathrm{norm}}$ represents the mean energy of water-water interaction in bulk region. The value of $\Delta H_{k}^{\mathrm{norm}}$ represents the mean interaction of a water molecule with the protein and all other water molecules, referenced to that of bulk, $2E_{\mathrm{bulk},\mathrm{ww}}^{\mathrm{norm}}$. Similarly, the total entropy of a water molecule in voxel k, relative to bulk, is defined as:

(2) $$-T\Delta S_{k}^{\mathrm{norm}}=-T\Delta S_{k,\mathrm{orient}}^{\mathrm{norm}}-T\Delta S_{k,\mathrm{trans}}^{\mathrm{norm}}\;$$

where T is the absolute temperature (that is included in the entropy terms of GIST by default). Accordingly, the free energy of a water molecule in voxel k, relative to bulk, is the sum of total enthalpy and entropy written as:

(3) $$\Delta G_{k}^{\mathrm{norm}}=\Delta H_{k}^{\mathrm{norm}}-T\Delta S_{k}^{\mathrm{norm}}\;.$$

Then, the unfavorable water molecule has a positive free energy ($\Delta G_{k}^{\mathrm{norm}}>0)$; in contrast, the favorable water molecule has a negative free energy ($\Delta G_{k}^{\mathrm{norm}}<0)$. As mentioned above, these thermodynamic quantities represent the differences from those of bulk water, which means that the displacement of high free-energy water is considered to be a driving force of protein-ligand binding.

### 2.2. AutoDock4

Our present method incorporates the GIST result into AutoDock4. AutoDock is one of the most widely used docking programs which is capable of quickly and accurately predicting bound conformation and binding energies [53]. In addition, AutoDock is widely used as a platform for the development of novel docking methodologies [48,58,59,60]. Two essential components of a docking program are an efficient search algorithm to find the conformation of the binding ligand and an accurate scoring function to estimate the binding free energy. AutoDock4 employs Lamarckian Genetic Algorithm (LGA) [61] for search algorithm and AutoDock4.2 force field [49] for the scoring function. The scoring function of AutoDock4 is a semiempirical free-energy force field written as:

(4) $$\begin{array}{cc} \Delta G_{\mathrm{bind}}^{\mathrm{AutoDock}} & =\Delta H_{\mathrm{vdW}}+\Delta H_{\mathrm{hbond}}+\Delta H_{\mathrm{elec}}+\Delta S_{\mathrm{conf}}+\Delta G_{\mathrm{desolv}}\\ & =W_{\mathrm{vdW}}\overset{\mathrm{lig}}{\underset{i}{\sum }}\overset{\mathrm{prot}}{\underset{j}{\sum }}\left(\frac{A_{ij}}{r_{ij}^{12}}-\frac{B_{ij}}{r_{ij}^{6}}\right)\\ & +W_{\mathrm{hbond}}\overset{\mathrm{lig}}{\underset{i}{\sum }}\overset{\mathrm{prot}}{\underset{j}{\sum }}E\left(\theta \right)\left(\frac{C_{ij}}{r_{ij}^{12}}-\frac{D_{ij}}{r_{ij}^{10}}\right)\\ & +W_{\mathrm{elec}}\overset{\mathrm{lig}}{\underset{i}{\sum }}\overset{\mathrm{prot}}{\underset{j}{\sum }}\left(\frac{q_{i}q_{j}}{\varepsilon \left(r_{ij}\right)r_{ij}}\right)\\ & +W_{\mathrm{conf}}N_{\mathrm{tor}}\\ & +W_{\mathrm{desolv}}\overset{\mathrm{lig}}{\underset{i}{\sum }}\overset{\mathrm{prot}}{\underset{j}{\sum }}\left(S_{i}V_{j}+S_{j}V_{i}\right)e^{\left(-r_{ij}^{2}/2\sigma^{2}\right)}\;. \end{array}$$

Here, the scoring function consists of five potential energy terms, including van der Waals $\Delta H_{\mathrm{vdW}}$, hydrogen bonding $\Delta H_{\mathrm{hbond}}$, electrostatic $\Delta H_{\mathrm{elec}}$, the conformational entropy of ligand $\Delta S_{\mathrm{conf}}$, and desolvation $\Delta G_{\mathrm{desolv}}$. The intermolecular potentials are calculated by summation over all pairs of ligand atom i and protein atom j as the function of their distance. The van der Waals term is a typical Lennard-Jones 12-6 dispersion/repulsion potential. The parameters A and B are taken from Amber force field [62]. The hydrogen bonding term is a Lennard-Jones 12-10 dispersion/repulsion potential with the directionality of hydrogen bond E(θ) depending on the angle θ and the parameters C and D [63]. The electrostatic term is a screened Coulomb potential with the distance-dependent dielectric function $\varepsilon \left(r_{ij}\right)$ [64]. The conformational entropy term represents the loss of torsional entropy upon binding, depending on the number of rotatable bonds of ligand $N_{\mathrm{tor}}$. The last term is a desolvation potential based on the volume V of atoms that surround a given atom and shelter it from the solvent, weighted by the charge-based solvation parameter S and the exponential term with distance-weighting factor σ [65]. The coefficients W are weight factors fitted using the training set of the crystal structure of protein-ligand complexes and the experimentally measured binding affinities. Since the scoring function of AutoDock4 has these weight factors, it is called a semiempirical scoring function.

**Figure 2:**
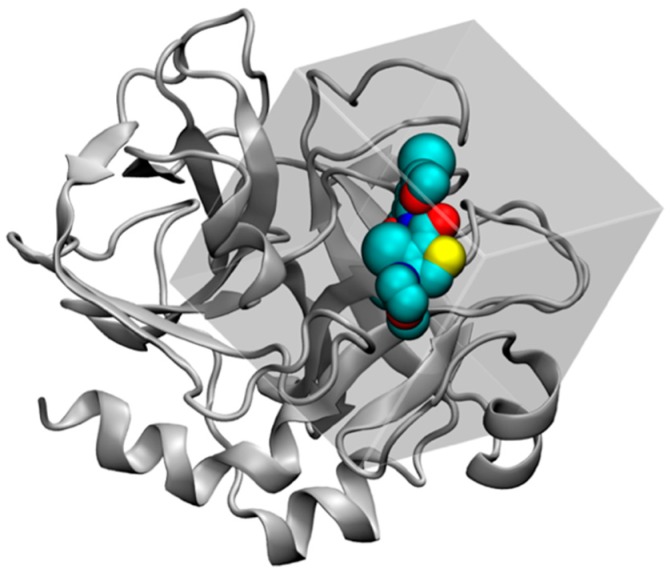
Co-crystal structure of FXa (cartoon) with ligand, Protein Data Bank (PDB) ligand-id (HET) XLD, in van der Waals representation (cyan). The cubic region represents the docking site of AutoDock4 (gray). Figure prepared by using VMD [57].

Using this scoring function and the optimization algorithm, AutoDock4 searches the most stable (i.e., the lowest energy) binding conformation of the ligand in the user-defined cubic docking site (Figure 2). To enable searching for a large conformational space available to a ligand in protein, AutoDock4 introduced a grid-based energy calculation method. In this approach, the binding site of a target protein is embedded in the grid map. Before the docking simulation, a probe atom is sequentially set on each grid center, and the interaction energy between a probe atom and the target protein is calculated and stored in the grid map. This grid map is used as a lookup table during the docking simulation for rapid energy evaluation of ligand conformations. This cubic docking region and grid-based potential calculation approach are quite suitable to be combined with the description of water properties by GIST.

### 2.3. Development of GIST-Based Desolvation Function and Its Incorporation into AutoDock4

Although the free energy change of displacing water can be calculated by GIST results directly, many previous studies reported that there was no direct correlation between the free energy of water molecules in the binding site and the affinity of bound ligands and that the use of the simplified scoring function performed well [12,30,38]. Hence, we developed GIST-based desolvation function according to a simple physical principle: If a heavy atom of ligand displaced a high-occupancy and unfavorable water molecule, the ligand earned a favorable contribution in binding free energy. The unfavorable water in this context corresponds to the high free-energy water for which the enthalpy-entropy compensation breaks down and either enthalpy or entropy is significantly unfavorable. Based on this physical principle, we design and propose a desolvation function suitable for grid-based energy calculation of AutoDock4. Once running MD simulation of apoprotein and explicit water and calculating thermodynamics of water with GIST, the grid water properties are readily converted to the map of unfavorable water according to two criteria as follows: (I) The free energy of a water molecule in a voxel k, $\Delta G_{k}^{\mathrm{norm}}$, is higher than a cutoff value $\Delta G_{\mathrm{co}}$; (II) The number density of a water molecule in a voxel k, $\rho_{k}$, is greater than a cutoff value $\rho_{\mathrm{co}}$. Using this map of unfavorable water, the displacing gain of an unfavorable water molecule is calculated as:

(5) $$\Delta G_{\mathrm{watdisp}}=\;\overset{\mathrm{lig}}{\underset{i}{\sum }}\delta_{i}\Delta G_{\mathrm{aff}}\;$$

(6) $$\delta_{i}=\{\begin{array}{cc} 1 & \mathrm{if}\;\mathrm{the}\;\mathrm{vdW}\;\mathrm{radius}\;\mathrm{of}\;a\;\mathrm{ligand}\;\mathrm{atom}\;i\;\mathrm{covers}\;\mathrm{unfavorable}\;\mathrm{water}\;\mathrm{grid}\;k\\ 0 & \mathrm{otherwise} \end{array}\;$$

**Figure 3:**
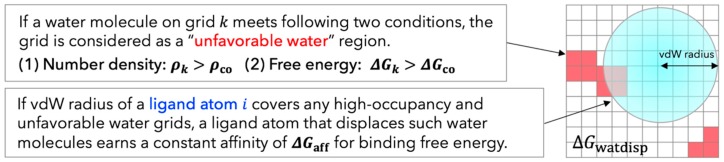
Diagram of GIST-based desolvation function employed here. A ligand atom i and unfavorable water grid k are represented by a blue sphere and red cubes, respectively.

Here, $\Delta G_{\mathrm{aff}}$ is a fitting parameter which specifies the free energy gain by displacement of the unfavorable water molecule; $\delta_{i}$ is a binary displacement indicator which equals 1 if the vdW radius of a ligand atom i covers any unfavorable water grid k and 0 otherwise. Figure 3 shows the diagram of this method. Note that our proposed method has three parameters, $\rho_{\mathrm{co}}$, $\Delta G_{\mathrm{co}}$, and $\Delta G_{\mathrm{aff}}$, which have to be fitted according to the binding thermodynamics of ligands.

The GIST-based solvation function $\Delta G_{\mathrm{watdisp}}$ was incorporated into the scoring function of AutoDock4, which is called AutoDock-GIST. This incorporation is achieved by a simple summation of the AutoDock4.2 force field and the GIST-based solvation function, expressed as:

(7) $$\Delta G_{\mathrm{bind}}^{\mathrm{AutoDock}-\mathrm{GIST}}=\Delta G_{\mathrm{bind}}^{\mathrm{AutoDock}}+\Delta G_{\mathrm{watdisp}}\;$$

Note that we retained the original desolvation term $\Delta G_{\mathrm{desolv}}$ of AutoDock4 (Equation (4)) in the proposed scoring function. Since the desolvation term $\Delta G_{\mathrm{desolv}}$ is based on the continuous solvation model and represents a penalty of binding free energy [49], we assumed that the displacing gain of unfavorable water $\Delta G_{\mathrm{watdisp}}$ does not conflict with $\Delta G_{\mathrm{desolv}}$. The AutoDock-GIST approach takes advantage of on-the-fly evaluation to search binding conformations in the docking process, as compared with other rescoring-after-docking models [66,67,68]. Since AutoDock uses the optimization algorithm to search the binding poses of ligand, the scoring function significantly affects the conformations of sampled binding poses. The pre-configured scoring function of AutoDock-GIST is capable of docking the ligand while taking into account the displacement of unfavorable water molecules. Furthermore, once calculating the GIST-based desolvation function, the AutoDock-GIST calculation can be implemented in high-throughput docking with almost the same computational cost as in AutoDock4.

The three fitting parameters of proposed scoring function, $\rho_{\mathrm{co}}$, $\Delta G_{\mathrm{co}}$, and ${\Delta G}_{\mathrm{aff}}$, were adjusted and validated using 51 ligands of FXa consisting of 28 training set ligands and 23 test set ligands. In this work, we sought two sets of optimal parameters for protein-ligand docking: (I) Affinity parameter set, which maximized the correlation between calculated score $\Delta G_{\mathrm{bind}}^{\mathrm{AutoDock}-\mathrm{GIST}}$and experimentally measured binding affinity $\Delta G_{\exp}$; (II) Pose parameter set, which maximized the success rate of binding pose prediction yielding root mean square deviation (RMSD) between docking pose and native pose of ligands less than 2 Å. To find these parameters, we scanned the value of $\rho_{\mathrm{co}}$ from 1.0 to 6.0 by increments of 0.1, the value of $\Delta G_{\mathrm{co}}$ from 0.0 to 4.0 kcal/mol by increments of 0.1 kcal/mol, and the value of $\Delta G_{\mathrm{aff}}$ from 0.0 to −2.0 kcal/mol by decrements of 0.01 kcal/mol, respectively. This scan yields 61×41×201=50,271 combinations of the three parameters. For each combination, the training set ligands are calculated with AutoDock-GIST, and evaluated by each of the two conditions above. The optimal parameters found in this procedure were then validated using the test set ligands.

### 2.4. Datasets and Preparation

#### 2.4.1. Structure Preparation and MD Simulation for FXa

In this work, we studied the coagulation factor Xa (FXa) to assess the performance of AutoDock-GIST. To analyze thermodynamics of active-site water molecules of FXa by GIST, we performed MD simulation of apoprotein and explicit surrounding water. The crystal structure of FXa was obtained from Protein Data Bank (PDB) [69] entry 1FJS [70], as studied previously [30,38]. First, we removed all crystallographic water molecules and bound ligands, keeping ions, from the system, and added hydrogens using the program Reduce [71]. We also removed the chain L of the crystal structure. We then used Tleap program from AmberTools [72] to prepare the system. We assigned protein parameters from AMBER99SB force field [73] and solvated the system in a TIP3P [74] water box with the periodic boundary condition, keeping the minimum distance of 10 Å away from any atom of the protein. Four disulfide bonds were set up and two crystal ions, Ca^2+^ and Cl^−^, were restrained at their original positions.

After preparing the system, we minimized the energy of the system and ran MD simulation. All following procedures were carried out with the Amber 14 software using pmemd.cuda [75]. First, we minimized the system energy in two steps: (I) Only the water while restraining all protein atoms; (II) The water and the protein hydrogen atoms while restraining the protein heavy atoms. Both minimization steps used 1500 cycles of the steepest descent algorithm followed by the conjugate gradient method for the maximum of 20,000 cycles, where the atoms were harmonically restrained with force constant of 100 kcal/mol/Å. Next, the system was heated for 200 ps from 0 K to 50 K in the NVT ensemble with the first simulation and the temperature was incremented by 50 K for 200 ps in the NPT ensemble until 300 K was reached. The system was then equilibrated for 10 ns at 300 K in the NPT ensemble. At the final volume, the system was equilibrated again for 5 ns at 300 K in the NVT ensemble. The final production MD run of 100 ns was performed in the NVT ensemble, and snapshots of this simulation were saved every 1 ps, for a total 10,000 frames of snapshots stored. Notably, during all MD simulations, all protein atoms were harmonically restrained with a force constant of 100 kcal/mol/Å. A time step of 2 fs was employed with SHAKE algorithm [76]. The temperature was regulated by Langevin thermostat; the nonbonded interactions were truncated at 9 Å and the particle mesh Ewald method was implemented to account for the long-range electrostatic interaction [77]. After all, for the GIST calculation, the trajectory of production MD was aligned across all frames referenced to the initial position of the protein, using the cpptraj program [78].

#### 2.4.2. GIST Calculation and Docking Set-up

Before the GIST calculation, we prepared the FXa structure for docking simulation, following the standard AutoDock protocol. First, the protein structure of 1FJS was aligned to the initial coordinate of MD trajectory, to superpose the GIST region and docking region of AutoDock. The bound ligand, water, and ions were removed from the system and polar hydrogens were added to the protein using AutoDockTools [53]. The docking site was set to 22.5×22.5×22.5 Å^3^ cubic region centered at bound ligand of 1FJS, which was the range to cover the active site of FXa. In this docking site, grid-based potential maps were calculated by AutoGrid (included in AutoDock suit). We then used a default grid size of 0.375 Å (approximately a quarter of the vdW radius of carbon atom) to calculate the grid-based potential maps of AutoDock, which resulted in the number of grid points of map 60×60×60.

The GIST calculation was performed using cpptraj program included in AmberTools [78,79]. The cubic region of GIST analysis was set to the active site of FXa, corresponding to the docking region set above. The grid centroid position was the center of docking site. The grid size was 60×60×60. The voxel side length (grid spacing) was 0.375 Å, the same as default grid size of AutoDock4. The thermodynamic properties of active-site water molecules were then calculated by GIST using MD snapshots, and the free energies of water molecules were calculated based on Equations (1)–(3), and subsequently, the GIST-based desolvation function was adjusted using the training set described below.

#### 2.4.3. Dataset Preparation and Docking Metrics

For the evaluation of proposed method, we used diverse 51 ligands of FXa for which both experimentally measured binding affinities and X-ray crystal structures are known. The 51 ligands were grouped into the training set and the test set to optimize and validate fitting parameters of GIST-based desolvation function. First, for the training set, we used 28 ligands of FXa which were used in a previous computational study [30] (Table S1). Next, for the test set, we selected an additional 23 ligands of FXa from PDBbind 2007 refined set [80] (Table S2). Note that we then eliminated some FXa ligands from original PDBbind dataset, which have the adverse correlation between molecular weight and binding affinity (e.g., a ligand with low molecular weight but high binding affinity or a ligand with high molecular weight but low binding affinity), since with such ligands it is quite difficult to estimate the correct binding affinity by scoring functions of docking programs [81,82]. As a result, the correlations between molecular weight and binding affinity of the training set and the test set are 0.48 and 0.33 in R^2^ values, respectively. All ligands in the training set and the test set were carefully aligned on the initial structure of simulated protein (1FJS) and their energies were minimized by AMBER12:EHT force field using Molecular Operating Environment (MOE) [83]. In addition, we used a compound dataset of FXa obtained from the directory of useful decoys-enhanced (DUD-E) [55] to validate the virtual screening performance of AutoDock-GIST. The virtual screening dataset includes 793 active and 20418 decoy compounds of FXa. All of the ligands used in this study were prepared for docking simulation, by using AutoDockTools [53].

The capability of AutoDock-GIST was assessed in terms of binding affinity prediction, docking success rate, and virtual screening performance. First, the accuracy of binding affinity prediction was measured by the correlation between the calculated score of native pose ligand and the experimentally measured binding affinity, for the R^2^ value of Pearson correlation coefficient. Next, the docking calculation was performed 10 times for each ligand, and the lowest energy conformation was selected. The docking success rate was then calculated based on RMSD between the predicted binding pose and crystal pose of ligand. In this work, an RMSD of less than 2 Å was regarded as a success of binding pose prediction. At last, the performance of virtual screening was evaluated by area under the curve (AUC) of receiver operating characteristic (ROC) [84] and enrichment factor (EF) [85]. The ROC curve plots the true positive rate against the false positive rate of virtual screening results, and the context of AUC represents the area under the ROC curve. The range of the AUC is 0 to 1: the value 1 represents ideal virtual screening result, and the value 0.5 represents random selection. The enrichment factor is a characteristic of a rank-ordered list of a given first x% subset, calculated as:

(8) $$\mathrm{EF}\left(x\%\right)=\frac{{\mathrm{hits}}_{x}/N_{x}}{{\mathrm{hits}}_{t}/N_{t}}\;$$

where ${\mathrm{hits}}_{x}$ is the number of actives found in the first x% subset, $N_{x}$ is the total number of compounds at first x% subset; ${\mathrm{hits}}_{t}$ and $N_{t}$ are the total number of actives and the total number of compounds in the entire docked dataset, respectively. Therefore, EF(x%) estimates how many times a docking program can pick out actives relative to random, in the first x% subset of a rank-ordered docking result.

## 3. Results and Discussion 

### 3.1. Parameter Fitting for GIST-Based Desolvation Function

In this section, we discuss adjusted parameters of GIST-based desolvation function and the unfavorable water distributions in the active site of FXa. As mentioned above, we constructed the two sets of parameters: (I) The affinity parameter set which maximized the correlation between docking score and experimentally measured binding affinity; and (II) The pose parameter set which maximized the success rate of binding pose prediction in docking. The values of three parameters were systematically searched from the parameter space using 28 training set ligands of FXa. As a result, we found optimal values of $\rho_{\mathrm{co}}$, $\Delta G_{\mathrm{co}}$, and $\Delta G_{\mathrm{aff}}$ for each parameter set (Table 1), so that docking and scoring performances were significantly improved, as will be discussed in the following sections.

In both parameter sets, density cutoff parameters $\rho_{\mathrm{co}}$ have high values beyond 4, in other words, the unfavorable water region of GIST-based desolvation function has over four-fold higher density than that for bulk water. The value of $\rho_{\mathrm{co}}$ in the affinity parameter set is slightly greater than that in the pose parameter set. On the other hand, the value of free-energy cutoff parameter $\Delta G_{\mathrm{co}}$ in the affinity parameter set is approximately a half of that in the pose parameter set, that is, the affinity parameter set picks up less unfavorable water molecules than the pose parameter set. The displacing gain of unfavorable water, $\Delta G_{\mathrm{aff}}$, is two-fold higher in the affinity parameter set than that in the pose parameter set. In summary, the affinity parameter set gives high free-energy gain to the displacement of unfavorable water molecules, while the pose parameter set gives low free-energy gain to displacement of highly unfavorable water molecules.

**Figure 4:**
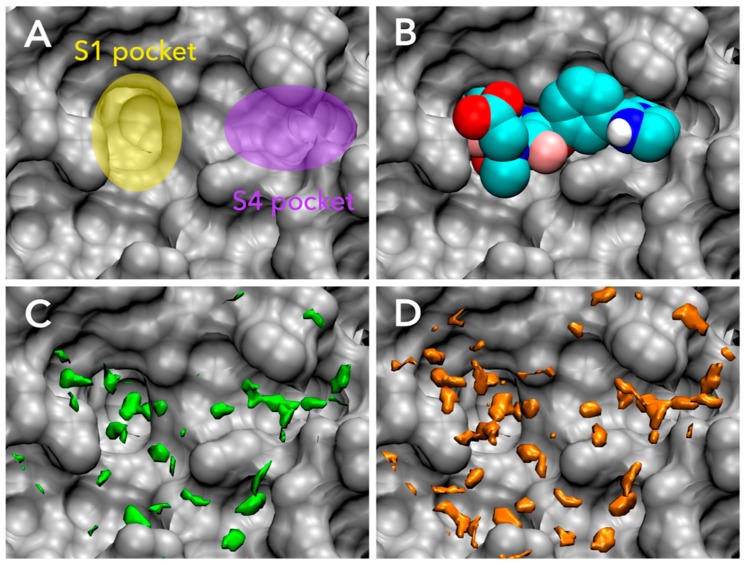
Binding ligand and distributions of unfavorable water for GIST-based desolvation function in the active site of FXa (PDB-id: 1FJS, gray): (**A**) Binding hot spots of FXa, S1 pocket (yellow), and S4 pocket (purple); (**B**) The bound ligand of 1FJS (residue-id: Z34), in van der Waals representation (cyan); (**C**) The unfavorable water distribution for pose parameter set (green); (**D**) The unfavorable water distribution for affinity parameter set (orange). Figure prepared by using VMD [57].

The active site of FXa and the distribution of unfavorable water for each parameter set are shown in Figure 4. The active site of FXa includes two important subpockets for bound inhibitors, S1 and S4 [86] (Figure 4A). The S1 pocket is a deeply concave region and determines the major component of selectivity and binding by residues Asp189, Ser195, and Tyr228. The S4 pocket, called hydrophobic box, is formed from three aromatic residues Tyr99, Phe174, and Trp215. FXa inhibitors are generally bound in an l-shaped conformation, where one group of the ligand occupies the anionic S1 pocket, and another group of the ligand occupies the aromatic S4 pocket; a fairly rigid linker group connects these two interaction sites [87]. The unfavorable water region of GIST-based desolvation function was determined by two cutoff parameters, density cutoff parameter $\rho_{\mathrm{co}}$ and free-energy cutoff parameter $\Delta G_{\mathrm{co}}$. In both parameter sets, the unfavorable water molecules were found in both S1 and S4 pockets; in other words, GIST analysis indicated that high-occupancy and high free-energy water molecules exist in S1 and S4 pockets. This result coincides with an early computational study of FXa by WaterMap [30]. However, the unfavorable water regions of the pose parameter set and affinity parameter set showed somewhat different configurations. For the pose parameter set, the high value of $\Delta G_{\mathrm{co}}$ caused the tight distribution of unfavorable water on the binding hot spots of FXa (Figure 4C). In contrast, for the affinity parameter set, the low value of $\Delta G_{\mathrm{co}}$ caused the broad water distribution covering the active-site surface of FXa (Figure 4D).

**Figure 5:**
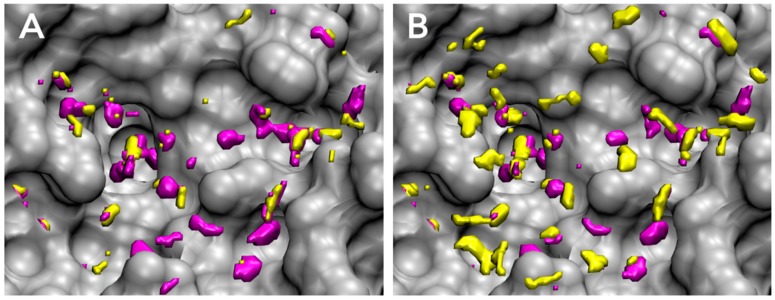
Enthalpy-entropy decomposition of unfavorable water distributions for GIST-based desolvation function in the active site of FXa (gray): (**A**) pose parameter set; (**B**) affinity parameter set. More enthalpically unfavorable water regions are shown in purple ($\Delta H_{k}^{\mathrm{norm}}>-T\Delta S_{k}^{\mathrm{norm}}$), whereas more entropically unfavorable water regions are shown in yellow ($\Delta H_{k}^{\mathrm{norm}}<-T\Delta S_{k}^{\mathrm{norm}}$). Figure prepared by using VMD [57].

For further discussion, we analyzed the free energy components of unfavorable waters in the active site of FXa. We have discussed the unfavorable active-site water in a term of high free energy so far. However, there are two types of unfavorable water regions which comprise enthalpically unstable water or entropically unstable water in an active-site of protein. It is widely known that enthalpy and entropy compensate each other in biomolecular systems [18,88,89,90]. For instance, a water molecule placed on a hydrophobic surface is enthalpically unfavorable, since it cannot make appropriate hydrogen bonds. However, at the same time, such water molecules are entropically favorable, because the missing hydrogen bond relaxes its orientation and earns orientational entropy. In contrast, a tightly bound water molecule is enthalpically favorable but entropically unfavorable due to its fixed orientation. The high free-energy water then causes the breakdown of enthalpy-entropy compensation and either enthalpy or entropy is significantly unfavorable. For each parameter set of GIST-based desolvation function, we decomposed unfavorable water region into an enthalpically unfavorable water and an entropically unfavorable water regions (Figure 5). Here, the enthalpically dominant water represents $\Delta H_{k}^{\mathrm{norm}}>-T\Delta S_{k}^{\mathrm{norm}}$, whereas the entropically dominant water represents $\Delta H_{k}^{\mathrm{norm}}<-T\Delta S_{k}^{\mathrm{norm}}$. The results showed that the unfavorable water for pose parameter set was more enthalpically unfavorable (Figure 5A), whereas that of affinity parameter set was more entropically unfavorable (Figure 5B). The main difference in two parameter sets was the value of free-energy cutoff $\Delta G_{\mathrm{co}}$: The value of $\Delta G_{\mathrm{co}}$ in the affinity parameter set is approximately a half of that in the pose parameter set. Hence, the results also indicate that the enthalpically unfavorable water is highly unfavorable in its free energy more than the entropically unfavorable water. In other words, the entropically unfavorable water is not so unfavorable in its free energy than the enthalpically unfavorable water.

### 3.2. Accuracy of Binding Affinity Prediction for FXa Ligands

**Figure 6:**
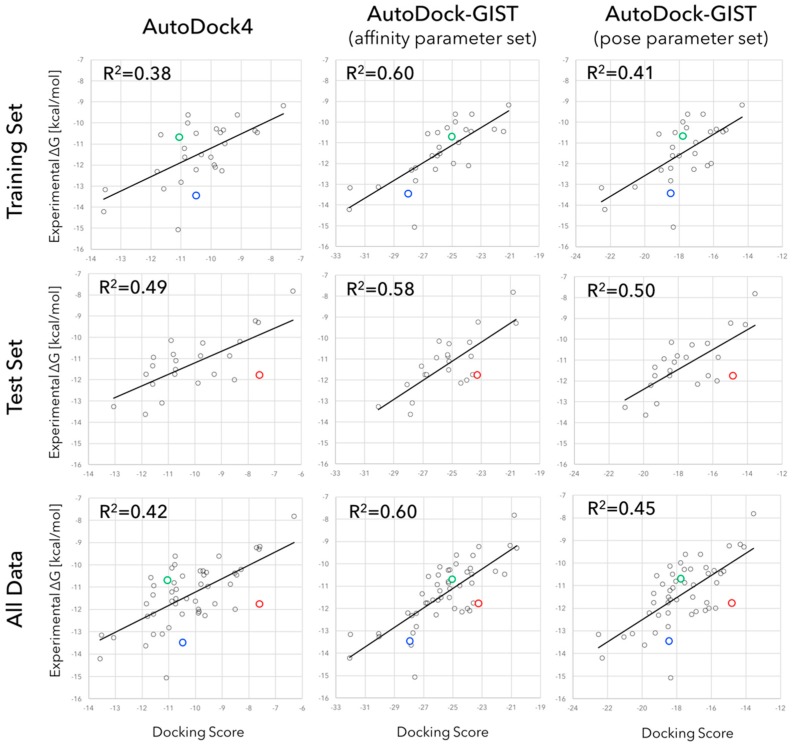
Scatter plots and regression lines of experimentally measured binding affinities versus docking scores of AutoDock4 (**left**), AutoDock-GIST with affinity parameter set (**middle**), and AutoDock-GIST with pose parameter set (**right**) for FXa ligands of training set (**upper**), test set (**middle**) and all data (**lower**). R^2^ values represent the squares of Pearson correlation coefficients. Color plots show specific examples of improvements: blue, green, and red circles represent the ligands of 1FJS, 2J34, and 2Y5F, respectively.

After the fitting parameters of GIST-based desolvation function were adjusted by 28 training sets ligands, the scoring accuracy of AutoDock-GIST was assessed for 23 test set ligands. Figure 6 shows the results of binding affinity predictions for FXa ligands. The R^2^ values between calculated score of AutoDock4 and experimentally measured binding affinity were 0.38 for the training set ligands and 0.49 for the test set ligands, respectively. In contrast, the affinity parameter set of AutoDock-GIST found the optimal parameters achieving the R^2^ value 0.60 for the training set ligands, and also improved the R^2^ value to 0.58 for the test set of ligands. Hence, this result has proved that the displacing gain of unfavorable water is an essential factor to improve the scoring function of docking. Some typical improvements are highlighted in Figure 6. For instance, AutoDock4 scoring function underestimated the binding free energy for the ligand of 1FJS (blue), since its interaction energy with the protein was not so high. On the other hand, the ligand of 1FJS successfully displaced some unfavorable water molecules and earned favorable free energy gain whose value of $\Delta G_{\mathrm{watdisp}}$ was −17.5 kcal/mol (Figure S1). A similar improvement was observed in the ligand of 2Y5F (red), which had poor interaction with the protein but displaced a great deal of unfavorable water (Figure S2). The value of $\Delta G_{\mathrm{watdisp}}$ was −16.0 kcal/mol for 2Y5F ligand. In contrast, the binding free energy of ligand of 2J34 (green) was overestimated by AutoDock4 scoring function, since it had favorable vdW interactions with protein atoms. However, the ligand of 2J34 earned little displacing gain of unfavorable water molecules so that the value of $\Delta G_{\mathrm{watdisp}}$ was −14.0 kcal/mol (Figure S3). As a result, these differences in the values of $\Delta G_{\mathrm{watdisp}}$ significantly improved the scoring accuracy of AutoDock-GIST with the affinity parameter set.

The same calculation was performed with the pose parameter set of AutoDock-GIST. Even though the pose parameter set was not adjusted in consideration of the accuracy of binding affinity prediction, interestingly, the R^2^ values were slightly improved, which are 0.41 and 0.50 for the training set and the test set, respectively. This result also supported the fact that the GIST-based desolvation function correctly described an essence of binding thermodynamics of ligand. On the other hand, since the pose parameter set had the higher value of free-energy cutoff $\Delta G_{\mathrm{co}}$ and the lower value of displacing gain $\Delta G_{\mathrm{aff}}$ than those of the affinity parameter set, the calculated result showed that these parameters did not significantly affect scoring accuracy. In other words, the result suggested that low free-energy cutoff value of unfavorable water and high displacing gain were effective for quantitative scoring of binding free energy.

The complete results of these computational experiments are available in Supplementary Materials (Tables S3 and S4). Notably, in this study, the absolute values of AutoDock-GIST scores were greater than those of AutoDock4 since we did not scale them in comparison to the experimental values.

### 3.3. Docking Success Rate for FXa Ligands

We expected that the displacement of unfavorable water molecules might contribute to the favorable conformation of binding ligand and inclusion of displacing gain should improve the docking performance. Based on this assumption, the pose parameter set of the GIST-based desolvation function was adjusted to be optimal for binding pose prediction using 28 training set ligands, and evaluated by 23 test set ligands. Table 2 shows the results of docking calculation of pose prediction success rates for FXa ligands. The docking success rates of AutoDock4 were 75.0% and 82.6% for the training set and the test set, respectively. As expected, the pose parameter set of AutoDock-GIST found the suitable parameters for binding pose prediction which resulted in a docking success rate 89.3% for the training set ligands, and also improved the docking success rate up to 95.7% for the test set ligands. On the other hand, for the affinity parameter set of AutoDock-GIST, the docking success rates were almost unchanged or were a little bit improved, which were 71.4% for the training set and 90.4% for the test set. The comprehensive results of docking calculations are shown in Supplementary Materials (Tables S5 and S6).

**Figure 7:**
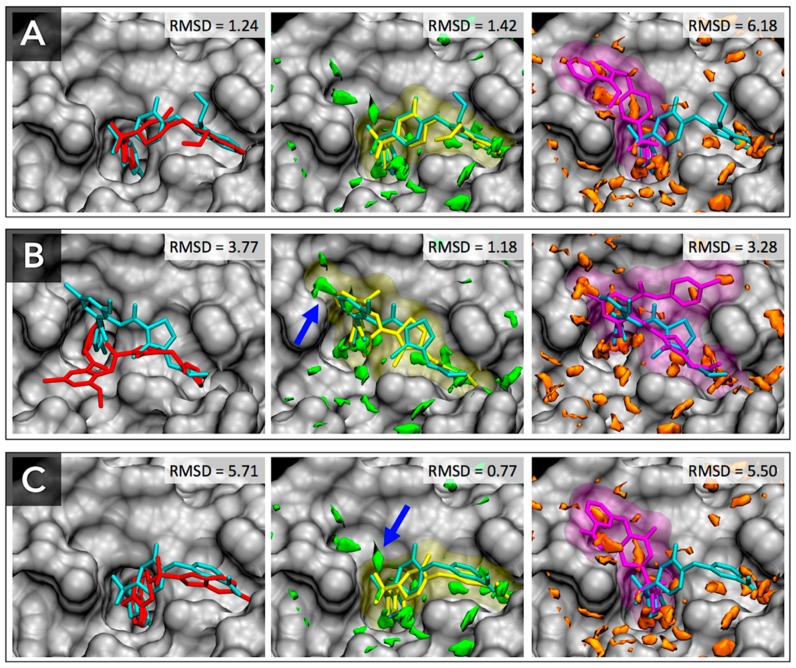
Docking results for FXa ligands of PDB entries: (**A**) 1NFX, (**B**) 1MQ6 and (**C**) 1NFU. Native crystallographic structures of bound ligands are shown as cyan sticks. Docking results of AutoDock4 are shown as red sticks (**left**), those of AutoDock-GIST with the pose parameter set are shown as yellow sticks and transparent surface (**middle**), and those of AutoDock-GIST with the affinity parameter set are shown as purple sticks and transparent surface (**right**). The unfavorable water distributions for the pose parameter set and the affinity parameter set are shown as green and orange regions, respectively. The blue arrows point to unfavorable water molecules which contribute to the successful docking. Figure prepared by using VMD [57].

For further discussion, we carefully analyzed docking results and found three typical cases that displacement of unfavorable water molecules affected conformations of docking ligands (Figure 7). First, for the ligand of 1NFX (residue-id: RDR), AutoDock4 successfully found the native-like pose of the bound ligand with RMSD of 1.24, and the pose parameter of AutoDock-GIST also reproduced the native-like pose of the bound ligand with RMSD of 1.42 (Figure 7A). However, the affinity parameter set of AutoDock-GIST failed to dock the ligand with RMSD of 6.18. In the other two cases for the ligands of 1MQ6 (residue-id: XLD) and 1NFU (residue-id: RRP), only the pose parameter set of AutoDock-GIST successfully reproduced the native-like poses, whereas AutoDock4 and the affinity set of AutoDock-GIST docked the ligands at far from native pose (Figure 7B,C). As mentioned above, the unfavorable water regions of the pose parameter set were mostly placed on the important binding pockets of FXa, S1, and S4 (Figure 4). The docking results clearly showed that the displacing gain of such unfavorable water molecules was an essential factor in determining the binding conformations of FXa ligands. In fact, the displacement of some unfavorable water in the pose parameter set indicated with blue arrows in Figure 7 seem to contribute to successful docking simulations. On the other hand, the affinity parameter set of AutoDock-GIST did not improve the docking success rate significantly, and found some unusual docking poses that were different from those of AutoDock4. In the cases of the affinity parameter set, we supposed possibilities that broad distribution of the unfavorable water and high displacing gain might yield unnecessary local minima in the free energy landscape of scoring function and merely caused docking failures.

### 3.4. Virtual Screening Performance of AutoDock-GIST

**Figure 8:**
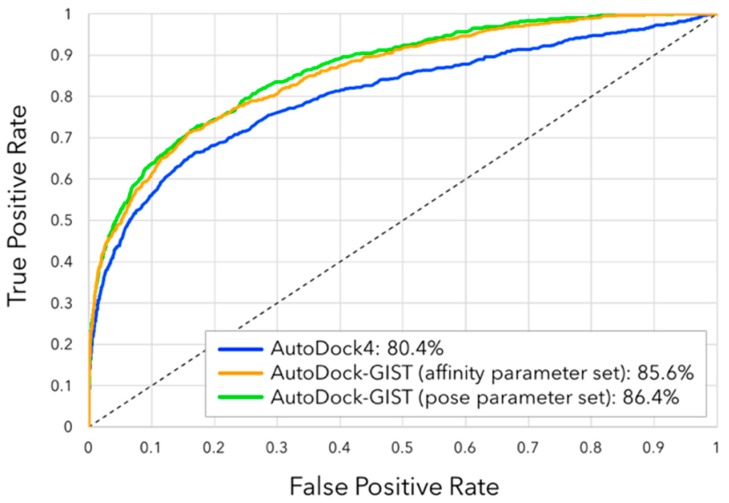
Receiver operating characteristic (ROC) plots of docking results for FXa ligands: AutoDock4 (blue), AutoDock-GIST with the affinity parameter set (orange), and AutoDock-GIST with the pose parameter set (green). The values represent the percentages of the AUC.

Another key measure of the docking performance is the enrichment of ligands among the top ranking docked compounds. We evaluated virtual screening performance of AutoDock-GIST through the docking calculation for 793 active and 20,418 decoy compounds of FXa from the directory of useful decoys-enhanced (DUD-E) [55]. Figure 8 shows the ROC plot and AUC of docking results. Though AutoDock4 showed good screening performance with AUC of 80.4%, both parameter sets of AutoDock-GIST improved the value of AUC compared with AutoDock4, which were 85.6% for the affinity parameter set and 86.4% for the pose parameter set. Interestingly, even though the pose parameter set of AutoDock-GIST was not adjusted in consideration of quantitative binding affinity of FXa ligands, it showed a slightly better performance than that of the affinity parameter set.

We also assessed the early enrichment of docking results by enrichment factor EF (Table 3). For all subset sizes, AutoDock-GIST resulted in superior performance to AutoDock4. The values 26.75 of EF(0.1%) in both parameter sets of AutoDock-GIST represent that 21 compounds of the top 0.1% subset were all active compounds, which are calculated by EF(0.1%)=(21/21)/(793/(20,418+793)) with Equation (8). The affinity parameter set of AutoDock-GIST showed the best value of EF(0.5), 25.23. For the larger subset, the pose parameter set of AutoDock-GIST performed the best. As mentioned above, the affinity parameter set of AutoDock-GIST tended to cause the docking failure frequently compared with that of the pose parameter set, and the incorrect binding pose then resulted in the wrong estimation of the binding affinity [91]. In other words, improvement of docking success rate with the pose parameter set contributed more positively to the virtual screening campaign than the affinity parameter set. Eventually, the virtual screening results indicated that our method was feasible to deal with diverse ligands of FXa and inclusion of displacing gain of unfavorable water molecules had a significant advantage in the docking campaign.

## 4. Conclusions

Although the thermodynamics of active-site water molecules are widely appreciated in the studies of molecular recognition, it is still challenging to estimate its contributions in protein-ligand docking quantitatively. Here, we showed a case study of the combination of GIST and AutoDock4, called AutoDock-GIST, and discussed the effectiveness of displacing gain of unfavorable water in protein-ligand docking. Following early key studies of GIST [38] and WaterMap [30], the present GIST-based desolvation function was designed on the basis of a simple physical principle: if a heavy atom of ligand displaced a high-occupancy and unfavorable water molecule, the ligand earned a favorable contribution in binding free energy. We studied diverse ligands of FXa by the proposed docking method and concluded that displacing gain of unfavorable water molecules was an essential factor for protein-ligand docking. The computational results showed that inclusion of water thermodynamics could improve not only quantitative scoring of binding affinity but also a conformational prediction of binding ligand. The result also indicated that the proposed method had a significant advantage in the virtual screening of the large compound set of FXa via docking.

Another interesting finding was that the high free-energy water molecules in the active site of FXa were mostly enthalpically unfavorable, rather than entropically unfavorable. This result is consistent with many previous studies that enthalpically unfavorable water molecules are more important for molecular recognition when they are displaced by a binding ligand [12,38]. In addition, our result revealed that the entropically unfavorable water molecules are also effective for quantitative binding affinity calculation when we consider the free energy of water molecules. However, our enthalpy dominant water model for the pose parameter set did not significantly improve the accuracy of binding affinity calculation. It implies that the displacement gain of enthalpically unfavorable water has a similar property to the scoring function of AutoDock4. It is possible that, since empirical or semiempirical scoring functions fit their interaction potentials to experimentally measured binding affinity ignoring the displacement of water molecules, they implicitly include a part of water replacement energies [92,93]. In fact, the weight factor of vdW potential in AutoDock4 scoring function is 1.37 times higher than that of hydrogen bonding potential. It might be modeling the difference of the displacement energy of active-site water molecules between a hydrophobically enclosed one (enthalpically unfavorable) and a hydrogen bonded one (enthalpically favorable). Hence, this presumption indicates that we should re-adjust potential parameters of scoring function with explicit water replacement terms for a more rigorous description of displacing water molecules.

Though the displacement of unfavorable water molecules is a principal driving force of the protein-ligand binding, it is worth mentioning that it is only a part of water thermodynamics upon ligand binding. For instance, some research groups reported that the displacement of tightly bound water molecules incurs a penalty in binding free energy [94,95,96]. It is also important to consider the contribution of hydrated water molecules, which remain and form a bridge of hydrogen bonds to proteins and binding ligands [2,97,98,99]. In both cases, GIST is capable of capturing such water molecules (Figures S4 and S5). However, an accurate modeling of water molecules becomes even more complicated in consideration of various thermodynamics of active-site water. It also needs a large dataset of protein-ligand complexes for further development of the scoring function because different protein binding site affect water differently so that a different result might be obtained for a different protein target. Some scoring functions attempted to cope with these kinds of challenging work, such as the WScore developed by Schrödinger, Inc., New York City, NY, USA [100]. Notably, while our work is a case study for a single target protein of FXa and further studies would be needed to show that this is a general result, our result supports the applicability of GIST for successful docking campaigns. We also hope that the present results would activate more quantitative studies of molecular docking for drug design.

## Figures and Tables

**Table 1:** Adjusted parameters for GIST-based desolvation function of AutoDock-GIST ^1^.

Parameter Set	$\rho_{\text{co}}$	$\Delta G_{\mathrm{co}}$ (kcal/mol/Water)	$\Delta G_{\mathrm{aff}}$ (kcal/mol)
Affinity parameter set	4.8	1.0	−0.50
Pose parameter set	4.3	1.9	−0.25

^1^ $\rho_{\mathrm{co}}$ is a density cutoff parameter for unfavorable water molecules in active site; $\Delta G_{\mathrm{co}}$ is a free-energy cutoff parameter for active-site water; $\Delta G_{\mathrm{aff}}$ is a free-energy gain of unfavorable water molecule displaced by a ligand heavy atom. Parameter fitting methods are described in the Materials and Methods section.

**Table 2:** Accuracies of binding pose predictions: docking success rates ^1^ for FXa ligands.

Data Set	AutoDock4	AutoDock-GIST (Pose Parameter Set)	AutoDock-GIST (Affinity Parameter Set)
Training set	75.0%	89.3%	71.4%
Test set	82.6%	95.7%	90.4%
All data	78.4%	92.1%	80.4%

^1^ RMSD between the native structure and the docking pose of ligand being less than 2 Å was regarded as a success of binding pose prediction.

**Table 3:** Enrichment factors for DUD-E ligands of FXa ^1^.

Metrics	AutoDock4	AutoDock-GIST (Affinity Parameter Set)	AutoDock-GIST (Pose Parameter Set)
EF(0.1%)	25.47	26.75	26.75
EF(0.5%)	23.22	25.23	24.73
EF(1%)	21.45	22.84	23.34
EF(2%)	16.65	17.92	19.11
EF(5%)	10.30	10.57	12.26
EF(10%)	6.44	6.61	7.59

^1^ The percentage in parenthesis represents the ratio of subset of the rank-ordered list in the docking result.

## Supplementary Materials

Supplementary materials can be accessed at: http://www.mdpi.com/1420-3049/21/11/1604/s1.

## Abbreviations

The following abbreviations are used in this manuscript:

SBDD	Structure-Based Drug Design
IST	Inhomogeneous Solvation Theory
GIST	Grid Inhomogeneous Solvation Theory
HAS	Hydration Site Analysis
MD	Molecular Dynamics
PDB	Protein Data Bank
RMSD	Root Mean Square Deviation
FXa	Factor Xa
DUD-E	Directory of Useful Decoys-Enhanced
ROC	Receiver Operating Characteristic
AUC	Area Under the Curve
EF	Enrichment Factor
