# Supplementary material for: AutoDock-GIST: Incorporating Thermodynamics of Active-Site Water into Scoring Function for Accurate Protein-Ligand Docking
Source: Molecules. 2016 Nov 23;21(11):1604. doi: 10.3390/molecules21111604 (PMC6274120; doi:10.3390/molecules21111604)
Supplement: Supplementary file 1 [file molecules-21-01604-s001.pdf]

Supplementary Materials: AutoDock-GIST: Incorporating Thermodynamics of Active-Site Water into Scoring Function for Accurate Protein-Ligand Docking

Shota Uehara and Shigenori Tanaka

Table S1. 28 ligands of coagulation factor Xa for the training set.

ID	PDB-ID/ res-ID	Chemical Structure	Compound Name	MW [g/mol]	ΔG_{exp} [kcal/mol] (Supplementary Reference)
1	1EZQ/RPR	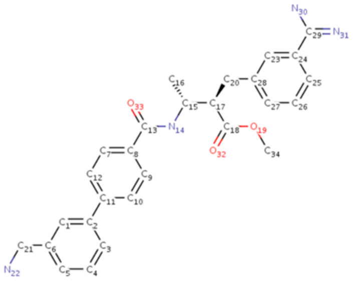	3-[(3′-Aminomethyl-biphenyl-4-carbonyl)-amino]-2-(3-carbamimidoyl-benzyl)-butyric acid methyl ester	458.5	−12.21 [1]
2	1F0R/815	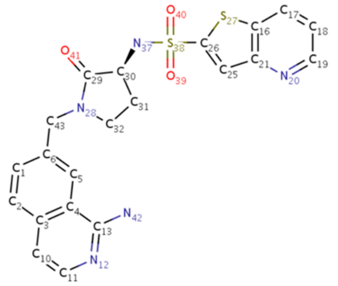	Thieno[3,2-b]pyridine-2-sulfonic acid [1-(1-amino-isoquinolin-7-ylmethyl)-2-oxo-pyrroldin-3-yl]-amide	453.5	−10.34 [2]
3	1F0S/PR2	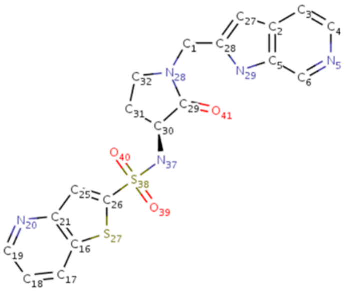	Thieno[3,2-b]pyridine-2-sulfonic acid [2-oxo-1-(1H-pyrrolo[2,3-c]pyridin-2-ylmethyl)-pyrrolidin-3-yl]-amide	427.5	−10.45 [2]
4	1FJS/Z34	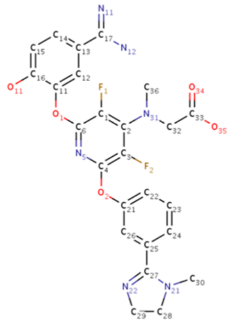	*N*-[2-[5-[Amino(imino)methyl]-2-hydroxyphenoxy]-3,5-difluoro-6-[3-(4,5-dihydro-1-methyl-1H-imidazol-2-yl)phenoxy]pyridin-4-yl]-*N*-methylglycine	526.5	−13.44 [3]

5	1G2L/T87	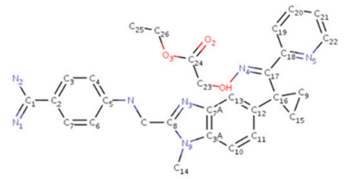	[(1-{2[(4-Carbamimidoyl-phenylamino)-methyl]-1-methyl-1H-benzoimidazol-5-yl}-cyclopropyl)-pyridin-2-yl-methyleneaminooxy]-acetic acid ethyl ester	525.6	−10.28 [4]
6	1G2M/R11	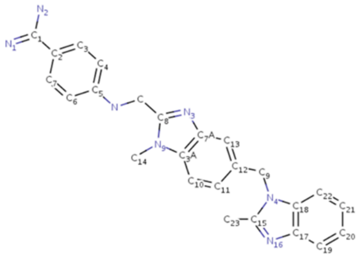	4-{[1-Methyl-5-(2-methyl-benzoimidazol-1-ylmethyl)-1H-benzoimidazol-2-ylmethyl]-amino}-benzamidine	423.5	−10.49 [4]
7	1KSN/FXV	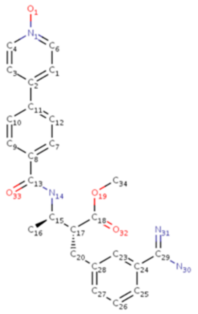	Methyl-3-(4′-N-oxopyridylphenoyl)-3-methyl-2-(m-amidinobenzyl)-propionate	447.5	−12.82 [5]
8	1LQD/CMI	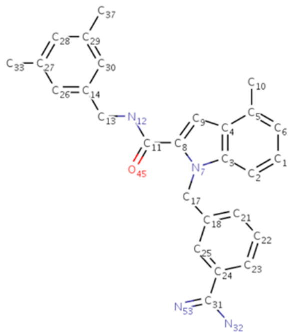	1-(3-Carbamimidoyl-benzyl)-4-methyl-1H-indole-2-carboxylic acid 3,5-dimethyl-benzylamide	424.5	−10.97 [6]
9	1MQ5/XLC	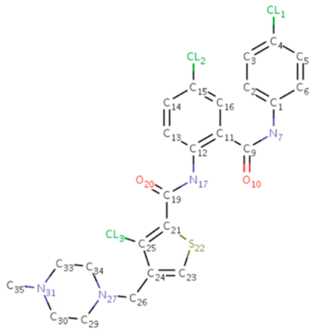	3-Chloro-*N*-[4-chloro-2-[[(4-chlorophenyl)amino]carbonyl]phenyl]-4-[(4-methyl-1-piperazinyl)methyl]-2-thiophenecarboxamide	537.9	−12.31 [7]

10	1MQ6/XLD	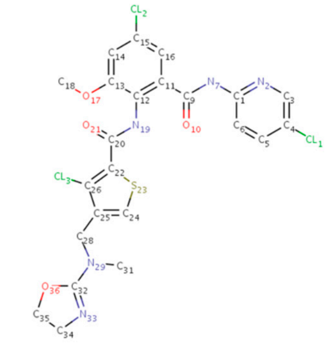	3-Chloro-*N*-[4-chloro-2-[[(5-chloro-2-pyridinyl)amino]carbonyl]-6-methoxyphenyl]-4-[[(4,5-dihydro-2-oxazolyl)methylamino]methyl]-2-thiophenecarboxamide	568.9	−15.06 [7]
11	1NFU/RRP	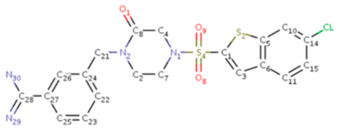	3-({4-[(6-Chloro-1-benzothien-2-yl)sulfonyl]-2-oxopiperazin-1-yl}methyl)benzenecarboximidamide	463.0	−10.45 [1]
12	1NFW/RRR	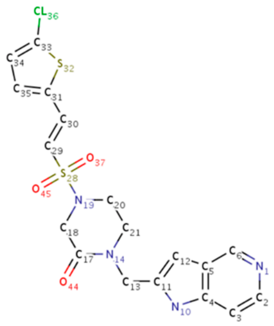	4-{[(e)-2-(5-Chlorothien-2-yl)vinyl]sulfonyl}-1-(1h-pyrrolo[3,2-c]pyridin-2-ylmethyl)piperazin-2-one	436.9	−12.09 [1]
13	1NFX/RDR	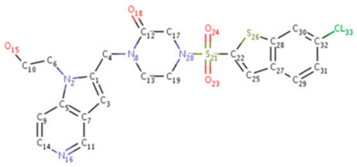	4-[(6-Chloro-1-benzothien-2-yl)sulfonyl]-1-{[1-(2-hydroxyethyl)-1H-pyrrolo[3,2-c]pyridin-2-yl]methyl}piperazin-2-one	505.0	−11.51 [1]
14	1NFY/RTR	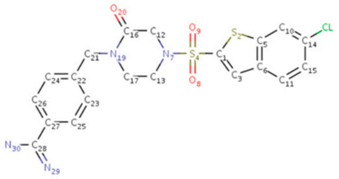	4-({4-[(6-Chloro-1-benzothien-2-yl)sulfonyl]-2-oxopiperazin-1-yl}methyl)benzenecarboximidamide	463.0	−12.00 [1]

15	1Z6E/IK8	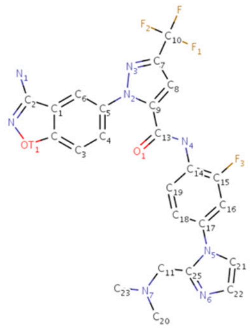	1-(3-Amino-1,2-benzisoxazol-5-yl)-*N*-(4-{2-[(dimethylamino)methyl]-1h-imidazol-1-yl}-2-fluorophenyl)-3-(trifluoromethyl)-1H-pyrazole-5-carboxamide	528.5	−13.12 [8]
16	2BMG/I1H	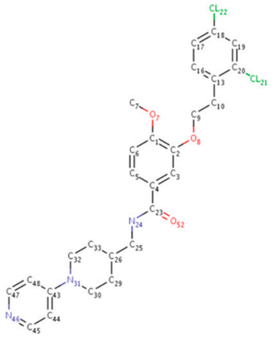	3-[2-(2,4-Dichlorophenyl)ethoxy]-4-methoxy-*N*-[(1-pyridin-4-ylpiperidin-4-yl)methyl]benzamide	514.4	−10.56 [9]
17	2BOH/IIA	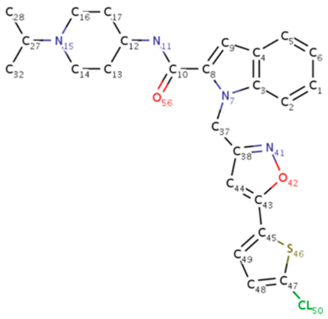	1-{[5-(5-Chloro-2-thienyl)isoxazol-3-yl]methyl}-*N*-(1-isopropylpiperidin-4-yl)-1h-indole-2-carboxamide	483.0	−11.62 [10]
18	2BOK/784	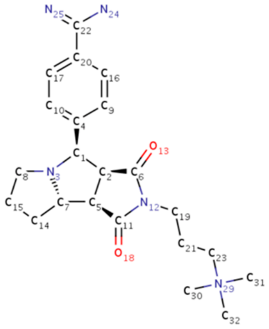	[Amino (4-{(3as,4r,8as,8br)-1,3-dioxo-2-[3-(trimethylammonio) propyl]decahydropyrrolo[3,4-a] pyrrolizin-4-yl}phenyl) methylene]ammonium	398.5	−9.173 [11]
19	2BQ7/IID	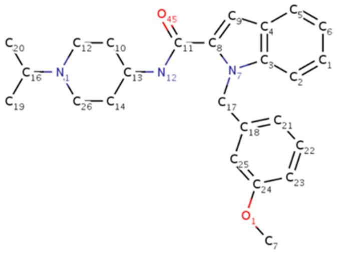	*N*-(1-Isopropylpiperidin-4-yl)-1-(3-methoxybenzyl)-1H-indole-2-carboxamide	405.5	−9.61 [10]

20	2BQW/IIE	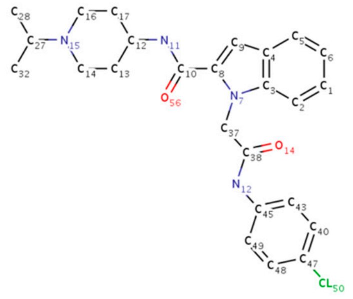	1-{2-[(4-Chlorophenyl)amino]-2-oxoethyl}-*N*-(1-isopropylpiperidin-4-yl)-1H-indole-2-carboxamide	453.0	−11.62 [10]
21	2CJI/GSK	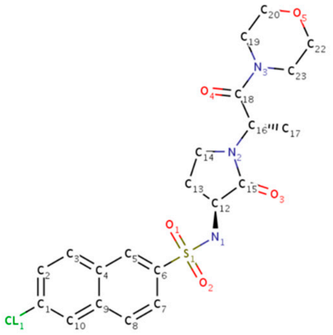	6-Chloro-*N*-{(3s)-1-[(1s)-1-methyl-2-(4-morpholinyl)-2-oxo ethyl]-2-oxo-3-pyrrolidinyl}-2-naphthalenesulfonamide	466.0	−11.21 [12]
22	2FZZ/5QC	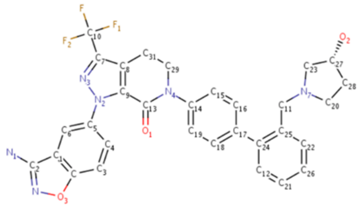	1-(3-Amino-1,2-benzisoxazol-5-yl)-6-(2′-{[(3r)-3-hydroxypyrrolidin-1-yl]methyl}biphenyl-4-yl)-3-(trifluoromethyl)-1,4,5,6-tetrahydro-7h-pyrazolo[3,4-c]pyridin-7-one	588.6	−14.21 [13]
23	2G00/4QC	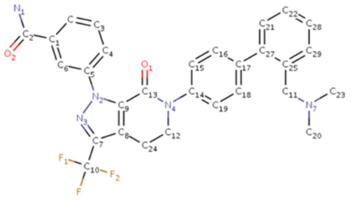	3-[6-{2′-[(Dimethylamino)methyl]biphenyl-4-yl}-7-oxo-3-(trifluoromethyl)-4,5,6,7-tetrahydro-1h-pyrazolo[3,4-c]pyridin-1-yl]benzamide	533.5	−13.16 [13]
24	2J2U/GSQ	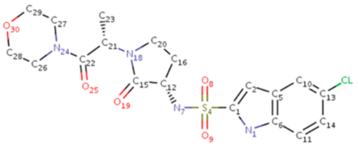	5-Chloro-*N*-{(3s)-1-[(1s)-1-methyl-2-morpholin-4-yl-2-5-chloro-n-{(3s)-1-[(1s)-1-methyl-2-morpholin-4-yl-2-sulfonamide	454.9	−9.61 [14]

25	2J34/GS6	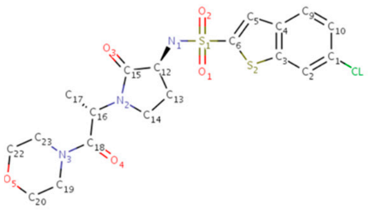	6-Chloro-*N*-{(3s)-1-[(1s)-1-methyl-2-morpholin-4-yl-2-oxoethyl]-2-oxopyrrolidin-3-yl}-1-benzothiophene-2-sulfonamide	472.0	−10.67 [14]
26	2J38/GS5	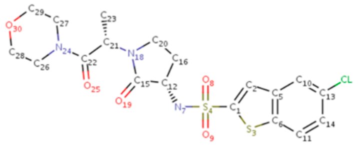	5-Chloro-*N*-{(3s)-1-[(1s)-1-methyl-2-morpholin-4-yl-2-oxoethyl]-2-oxopyrrolidin-3-yl}-1-benzothiophene-2-sulfonamide	472.0	−9.99 [14]
27	2J4I/GSJ	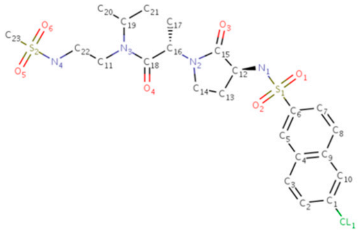	1-Pyrrolidineacetamide, 3-[[(6-chloro-2-naphthalenyl)sulfonyl]amino]-alpha-methyl-n-(1-methylethyl)-*N*-[2-[(methylsulfonyl)amino] ethyl]-2-oxo-, (alphas,3s)-	559.1	−12.27 [12]
28	3LIW/RUP	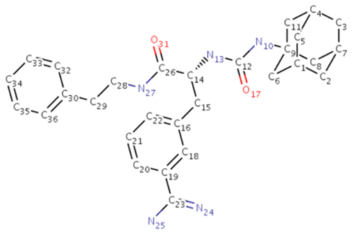	(r)-2-(3-Adamantan-1-yl-ureido)-3-(3-carbamimidoyl-phenyl)-*N*-phenethyl-propionamide	487.6	−10.37 [15]

Table S2. 23 ligands of coagulation factor Xa for the test set.

ID	PDB-ID/ res-ID	Chemical Structure	Compound Name	MW [g/mol]	ΔG_{exp} [kcal/mol] (Supplementary Reference)

1	1LPK/CBB	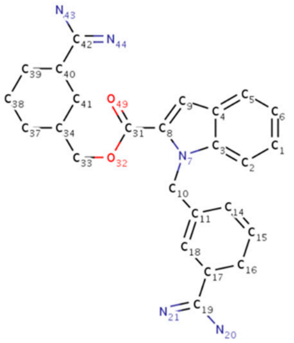	1-(3-Carbamimidoyl-benzyl)-1H-indole-2-carboxylic acid 3-carbamimidoyl-benzylester	433.6	−10.20 [6]
2	1LPZ/CMB	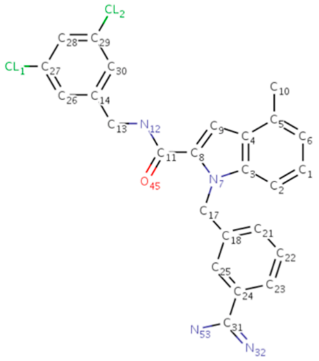	1-(3-Carbamimidoylbenzyl)-*N*-(3,5-dichlorobenzyl)-4-methyl-1h-indole-2-carboxamide	465.4	−10.26 [6]
3	1XKA/4PP	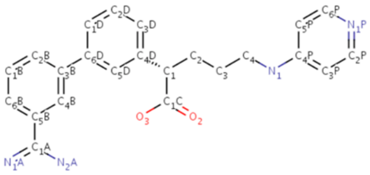	(2s)-(3′-Amidino-3-biphenyl)-5-(4-pyridylamino)pentanoic acid	388.5	−9.29 [16]
4	2JKH/BI7	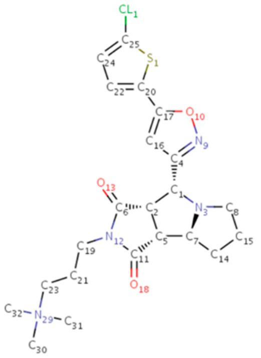	3-[(3as,4r,8as,8br)-4-[5-(5-chloro-2-thienyl)isoxazol-3-yl]-1,3-dioxooctahydropyrrolo[3,4-a]pyrrolizin-2(3h)-yl]-*N*,*N*,*N*-trimethylpropan-1-aminium	464.0	−10.86 [17]
5	2P16/GG2	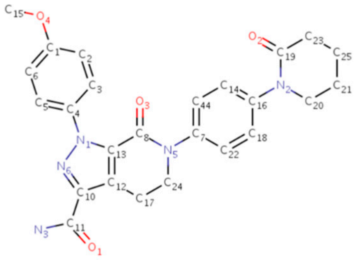	1-(4-Methoxyphenyl)-7-oxo-6-[4-(2-oxopiperidin-1-yl)phenyl]-4,5,6,7-tetrahydro-1h-pyrazolo[3,4-c]pyridine-3-carboxamide	459.5	−13.63 [18]

6	2VH6/GSV	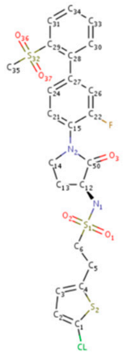	2-(5-Chlorothiophen-2-yl)-*N*-{(3s)-1-[3-fluoro-2′-(methylsulfonyl)biphenyl-4-yl]-2-oxopyrrolidin-3-yl}ethanesulfonamide	557.1	−13.09 [19]
7	2VVC/LZF	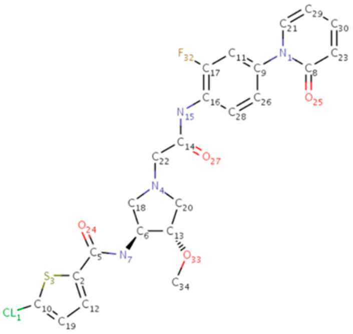	5-Chloro-*N*-[(3s,4s)-1-(2-{[2-fluoro-4-(2-oxopyridin-1(2H)-yl)phenyl]amino}-2-oxoethyl)-4-methoxypyrrolidin-3-yl]thiophene-2-carboxamide	505.0	−11.51 [20]
8	2VVU/H22	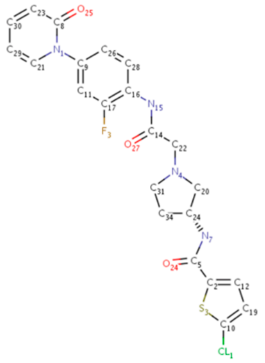	5-Chloro-*N*-[(3r)-1-(2-{[2-fluoro-4-(2-oxopyridin-1(2H)-yl)phenyl]amino}-2-oxoethyl)pyrrolidin-3-yl]thiophene-2-carboxamide	474.9	−10.93 [20]
9	2VVV/H21	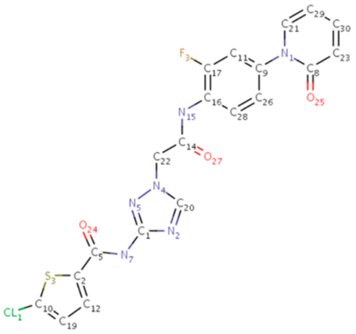	5-Chloro-*N*-[1-(2-{[2-fluoro-4-(2-oxopyridin-1(2H)-yl)phenyl]amino}-2-oxoethyl)-1H-1,2,4-triazol-3-yl]thiophene-2-carboxamide	472.9	−11.10 [20]
10	2VWN/H25	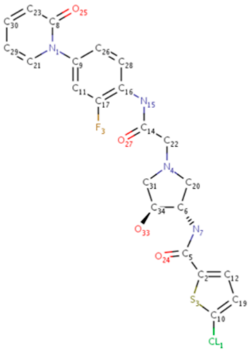	5-Chloro-thiophene-2-carboxylic acid ((3s,4s)-1-{[2-fluoro-4-(2-oxo-2h-pyridin-1-yl)-phenylcarbamoyl]-methyl}-4-hydroxy-pyrrolidin-3-yl)-amide	490.9	−10.80 [20]

11	2VWO/LZG	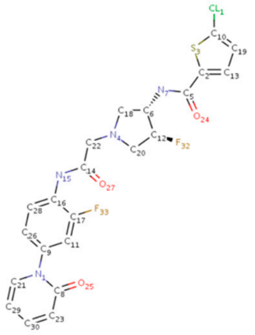	5-Chloro-thiophene-2-carboxylic acid ((3s,4s)-4-fluoro-1-{[2-fluoro-4-(2-oxo-2h-pyridin-1-yl)-phenylcarbamoyl]-methyl}-pyrrolidin-3-yl)-amide	492.9	−10.14 [20]
12	2WYG/461	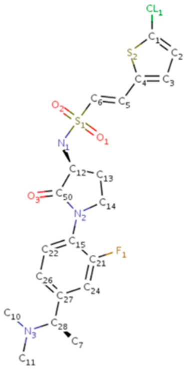	(e)-2-(5-Chlorothiophen-2-yl)-*N*-[(3s)-1-{4-[(1r)-1-(dimethylamino)ethyl]-2-fluorophenyl}-2-oxopyrrolidin-3-yl]ethenesulfonamide	472.0	−11.74 [21]
13	2WYJ/898	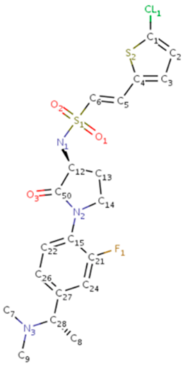	(e)-2-(5-Chlorothiophen-2-yl)-*N*-[(3s)-1-{4-[(1s)-1-(dimethylamino)ethyl]-2-fluorophenyl}-2-oxopyrrolidin-3-yl]ethenesulfonamide	472.0	−12.15 [21]
14	2Y5F/XWG	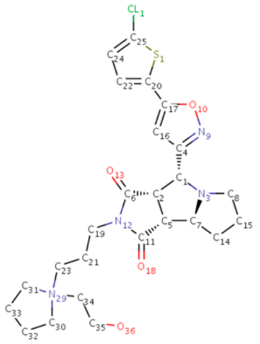	(3as,4r,5s,8as,8br)-4-[5-(5-Chlorothiophen-2-yl)-1,2-oxazol-3-yl]-2-[3-[1-(2-hydroxyethyl)pyrrolidin-1-ium-1-yl]propyl]-4,6,7,8,8a,8b-hexahydro-3ah-pyrrolo[3,4-a]pyrrolizine-1,3-dione	520.1	−11.74 [22]
15	2Y5G/FJD	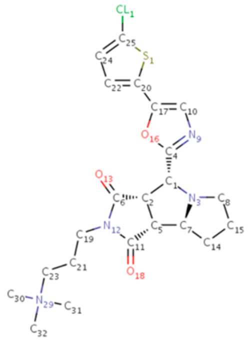	3-[(3as,4r,5s,8as,8br)-4-[5-(5-Chlorothiophen-2-yl)-1,3-oxazol-2-yl]-1,3-dioxo-4,6,7,8,8a,8b-hexahydro-3ah-pyrrolo[3,4-a]pyrrolizin-2-yl]propyl-trimethyl-azanium	464.0	−9.23 [22]

16	2Y5H/Y5H	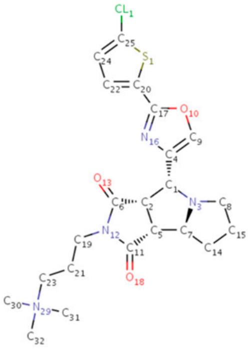	3-[(3as,4r,5s,8as,8br)-4-[2-(5-Chlorothiophen-2-yl)-1,3-oxazol-4-yl]-1,3-dioxo-4,6,7,8,8a,8b-hexahydro-3ah-pyrrolo[3,4-a]pyrrolizin-2-yl]propyl-trimethyl-azanium	464.0	−7.82 [22]
17	2Y7X/MZA	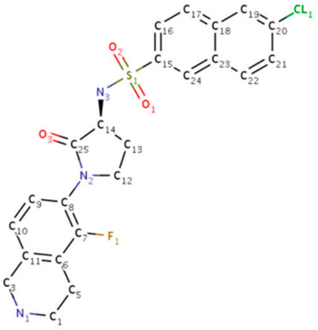	6-Chloro-*N*-[(3s)-1-(5-fluoro-1,2,3,4-tetrahydroisoquinolin-6-yl)-2-oxo-pyrrolidin-3-yl]naphthalene-2-sulfonamide	474.0	−12.00 [23]
18	2Y7Z/C0Z	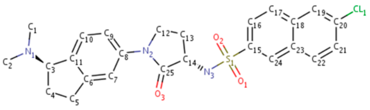	6-Chloro-*N*-[(3s)-1-[(1s)-1-dimethylamino-2,3-dihydro-1h-inden-5-yl]-2-oxo-pyrrolidin-3-yl]naphthalene-2-sulfonamide	484.0	−11.74 [24]
19	2Y80/439	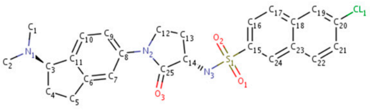	6-Chloro-*N*-{(3s)-1-[(1s)-1-(dimethylamino)-2,3-dihydro-1H-inden-5-yl]-2-oxo-3-pyrrolidinyl}-2-naphthalenesulfonamide	484.0	−10.86 [24]
20	2Y81/931	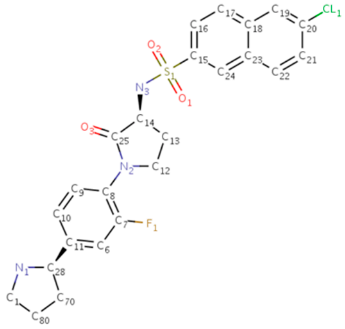	6-Chloro-*N*-((3s)-2-oxo-1-{4-[(2r)-2--pyrrolidinyl] phenyl}-3-pyrrolidinyl)-2-naphthalenesulfonamide	488.0	−11.74 [24]

21	2Y82/930	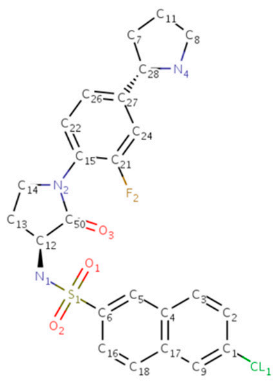	6-Chloro-*N*-((3s)-2-oxo-1-{4-[(2s)-2-pyrrolidinyl]phenyl}-3-pyrrolidinyl)-2-naphthalenesulfonamide	488.0	−11.34 [24]
22	3M36/M35	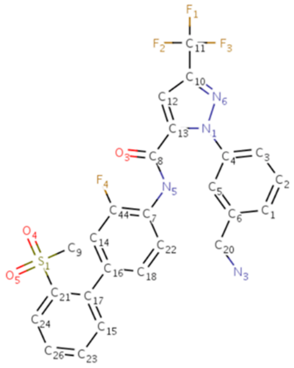	1-[3-(Aminomethyl)phenyl]-*N*-[3-fluoro-2′-(methylsulfonyl)biphenyl-4-yl]-3-(trifluoromethyl)-1H-pyrazole-5-carboxamide	532.5	−13.26 [8]
23	3M37/M37	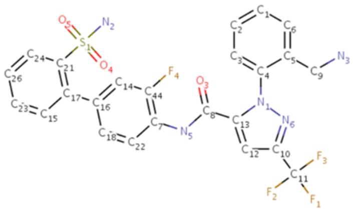	1-[2-(Aminomethyl)phenyl]-*N*-(3-fluoro-2′-sulfamoylbiphenyl-4-yl)-3-(trifluoromethyl)-1h-pyrazole-5-carboxamide	533.5	−12.21 [25]

Table S3. Experimentally measured binding free energies and calculated scores for the training set [1].

PDB-ID	ΔG$_{exp}$ [kcal/mol]	AutoDock4 Score	AutoDock-GIST Score [2] (Affinity Parameter Set)	AutoDock-GIST Score [2] (Pose Parameter Set)
1EZQ	−12.21	−10.50	−27.50	−18.50
1F0R	−10.34	−9.59	−22.09	−15.84
1F0S	−10.45	−8.46	−21.46	−15.46
1FJS	−13.44	−10.51	−28.01	−18.51
1G2L	−10.28	−9.82	−25.32	−17.57
1G2M	−10.49	−10.49	−25.99	−18.24
1KSN	−12.82	−11.00	−27.50	−18.50
1LQD	−10.97	−9.54	−24.54	−17.04
1MQ5	−12.31	−11.80	−27.80	−19.05
1MQ6	−15.06	−11.10	−27.60	−18.35
1NFU	−10.45	−9.69	−23.69	−16.19
1NFW	−12.09	−9.86	−23.86	−16.36
1NFX	−11.51	−10.33	−25.83	−17.08
1NFY	−12.00	−9.89	−24.89	−16.14
1Z6E	−13.12	−11.57	−30.07	−20.57
2BMG	−10.56	−11.67	−26.67	−19.17
2BOH	−11.62	−10.01	−26.01	−18.01
2BOK	−9.173	−7.59	−21.09	−14.34
2BQ7	−9.61	−9.12	−23.62	−16.62
2BQW	−11.62	−10.88	−26.38	−18.38
2CJI	−11.21	−10.89	−25.89	−18.39
2FZZ	−14.21	−13.57	−32.07	−22.32
2G00	−13.16	−13.52	−32.02	−22.52
2J2U	−9.61	−10.76	−24.76	−17.51
2J34	−10.67	−11.05	−25.05	−17.80
2J38	−9.99	−10.78	−24.78	−17.78
2J4I	−12.27	−9.64	−26.14	−17.14
3LIW	−10.37	−8.53	−24.03	−15.28

[1] Crystal structures of 28 binding ligands for coagulation factor Xa. [2] The absolute values of AutoDock-GIST scores are not scaled to those of experimentally measured binding free energies.

Table S4. Experimentally measured binding free energies and calculated scores for the test set [1].

PDB-ID	ΔG$_{exp}$ [kcal/mol]	AutoDock4 Score	AutoDock-GIST Score [2] (Affinity Parameter Set)	AutoDock-GIST Score [2] (Pose Parameter Set)
1LPK	−10.20	−8.30	−23.80	−16.30
1LPZ	−10.26	−9.71	−25.21	−17.21
1XKA	−9.29	−7.62	−20.62	−14.12
2JKH	−10.86	−8.70	−23.70	−15.70
2P16	−13.63	−11.87	−27.87	−19.87
2VH6	−13.09	−11.24	−27.74	−19.24
2VVC	−11.51	−10.73	−25.23	−18.48
2VVU	−10.93	−11.56	−26.06	−18.81
2VVV	−11.10	−10.72	−25.22	−18.22
2VWN	−10.80	−10.81	−25.31	−18.06
2VWO	−10.14	−10.89	−25.89	−18.39
2WYG	−11.74	−9.27	−23.27	−16.27
2WYJ	−12.15	−9.89	−24.39	−16.89
2Y5F	−11.74	−7.60	−23.60	−14.85
2Y5G	−9.23	−7.72	−23.22	−14.97
2Y5H	−7.82	−6.31	−20.81	−13.56
2Y7X	−12.00	−8.51	−24.01	−15.76
2Y7Z	−11.74	−10.75	−26.75	−18.50
2Y80	−10.86	−9.79	−25.29	−17.54
2Y81	−11.74	−11.84	−26.84	−19.34
2Y82	−11.34	−11.59	−27.09	−19.34
3M36	−13.26	−13.06	−30.06	−21.06
3M37	−12.21	−11.58	−28.08	−19.58

[1] Crystal structures of 23 binding ligands for coagulation factor Xa. [2] The absolute values of AutoDock-GIST scores are not scaled to those of experimentally measured binding free energies.

Table S5. RMSDs between crystal structures and docking poses for the training set.

PDB-ID	AutoDock4 [Å]	AutoDock-GIST (Affinity Parameter Set) [Å]	AutoDock-GIST (Pose Parameter Set) [Å]
1EZQ	1.47	1.78	1.15
1F0R	1.60	1.67	1.76
1F0S	1.71	1.66	1.83
1FJS	0.89	0.68	1.11
1G2L	1.05	1.06	1.17
1G2M	0.91	0.90	0.92
1KSN	1.64	0.87	0.70
1LQD	2.61	3.15	2.47
1MQ5	0.86	2.25	0.78
1MQ6	3.77	3.28	1.18
1NFU	5.71	5.50	0.77
1NFW	0.75	1.65	0.92
1NFX	1.24	6.18	1.42
1NFY	0.68	0.94	0.77
1Z6E	0.57	0.67	0.55
2BMG	3.70	1.24	3.02
2BOH	1.02	0.62	0.55
2BOK	0.58	1.34	0.51
2BQ7	2.90	0.52	1.17
2BQW	1.78	3.49	4.52
2CJI	1.09	2.78	1.03
2FZZ	1.47	0.55	1.25
2G00	0.63	2.89	0.31
2J2U	1.54	1.58	0.22
2J34	0.45	1.07	0.25
2J38	0.78	1.39	0.30
2J4I	3.72	0.36	1.85
3LIW	2.18	0.24	1.19

Table S6. RMSDs between crystal structures and docking poses for the test set.

PDB-ID	AutoDock4 [Å]	AutoDock-GIST (Affinity Parameter Set) [Å]	AutoDock-GIST (Pose Parameter Set) [Å]
1LPK	4.43	0.19	2.87
1LPZ	3.71	3.72	1.28
1XKA	3.06	0.66	0.96
2JKH	0.89	0.26	0.44
2P16	0.24	0.36	0.24
2VH6	1.81	1.38	0.90
2VVC	1.18	1.13	0.48
2VVU	1.02	1.03	0.39
2VVV	0.78	1.04	0.80
2VWN	1.33	0.97	0.39
2VWO	1.72	0.23	0.43
2WYG	1.33	1.22	1.55
2WYJ	1.05	0.95	1.18
2Y5F	1.22	0.62	1.14
2Y5G	0.61	2.73	0.55
2Y5H	2.75	1.11	0.84
2Y7X	1.20	0.30	1.15
2Y7Z	0.64	0.90	0.25
2Y80	0.71	0.39	0.65
2Y81	0.33	0.33	0.13
2Y82	0.43	1.53	0.31
3M36	0.77	0.58	0.61
3M37	1.86	1.79	1.90

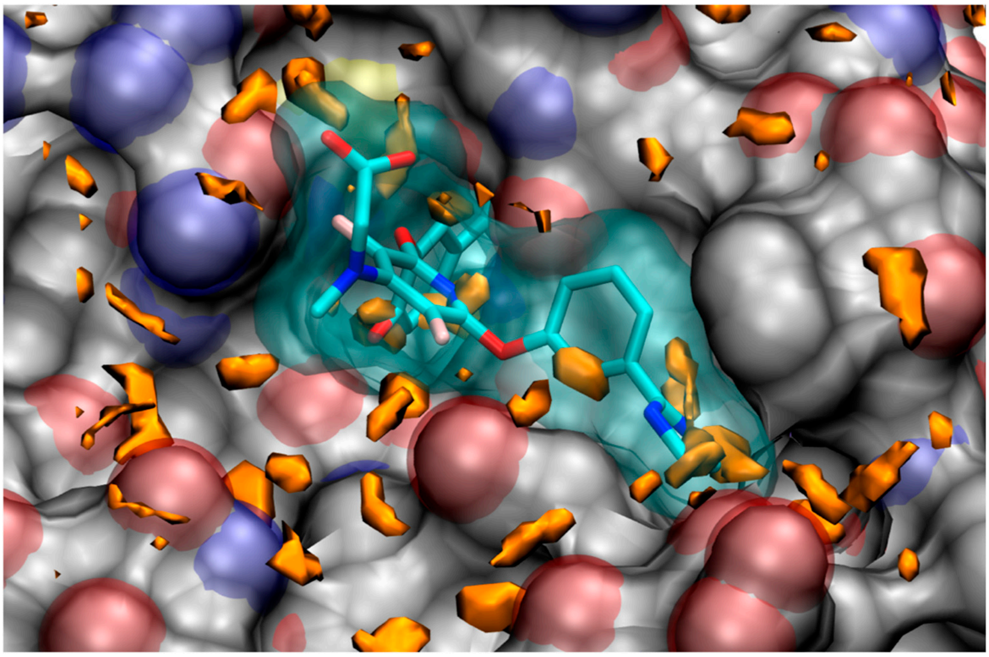

Figure S1. The structure of 1FJS ligand (cyan stick and transparent surface) bound to the active site of FXa (gray). Oxygen, nitrogen, sulfur and fluorine are represented in red, blue yellow and pink, respectively. The unfavorable water distribution of affinity parameter set is shown in orange.

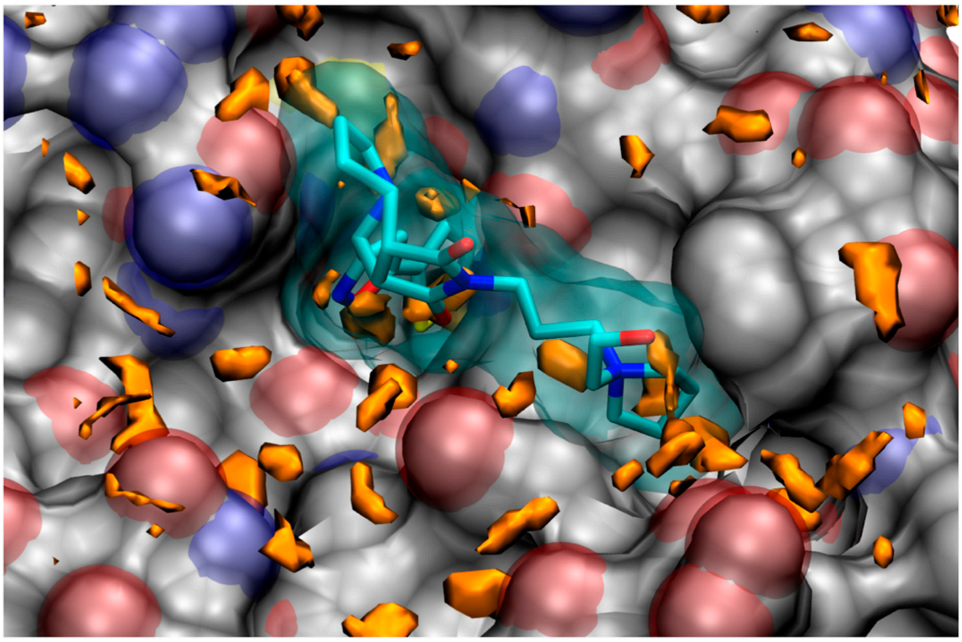

Figure S2. The structure of 2Y5F ligand (cyan stick and transparent surface) bound to the active site of FXa (gray). Oxygen, nitrogen and sulfur are represented in red, blue and yellow, respectively. The unfavorable water distribution of affinity parameter set is shown in orange.

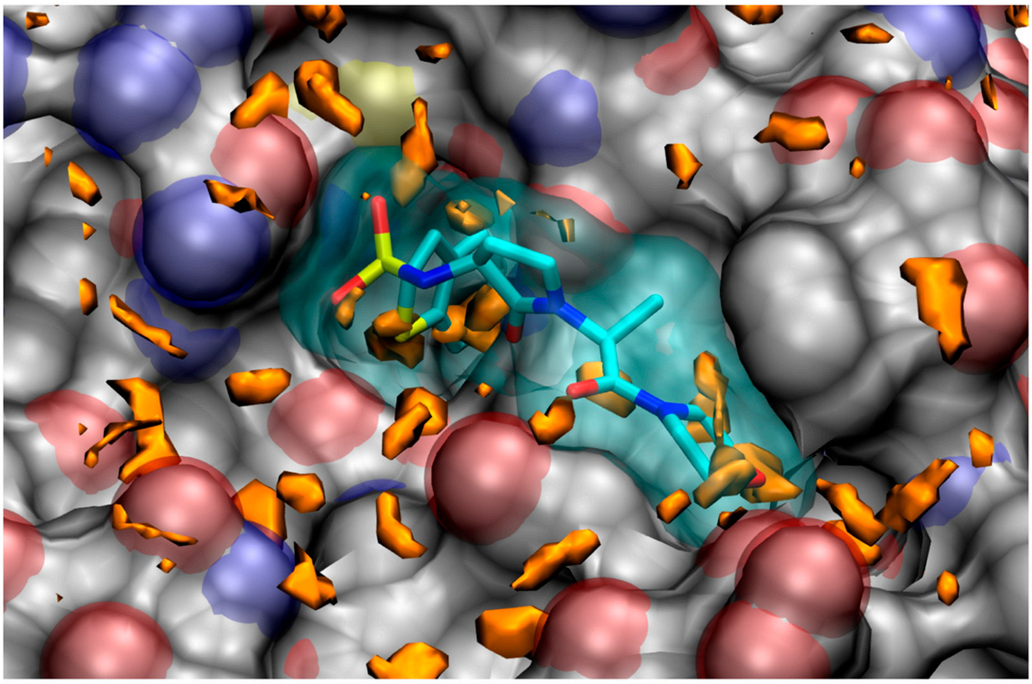

Figure S3. The structure of 2J34 ligand (cyan stick and transparent surface) bound to the active site of FXa (gray). Oxygen, nitrogen and sulfur are represented in red, blue and yellow, respectively. The unfavorable water distribution of affinity parameter set is shown in orange.

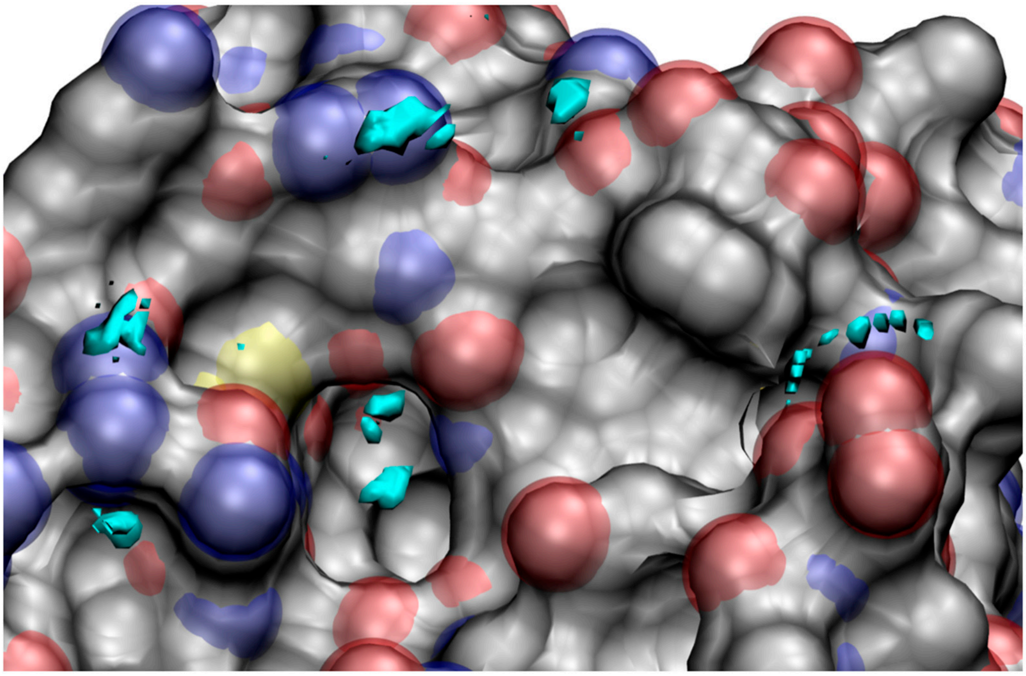

Figure S4. The high-occupancy and favorable water distribution (cyan) in FXa active site (gray) calculated by GIST. Oxygen, nitrogen and sulfur are represented in red, blue and yellow, respectively.

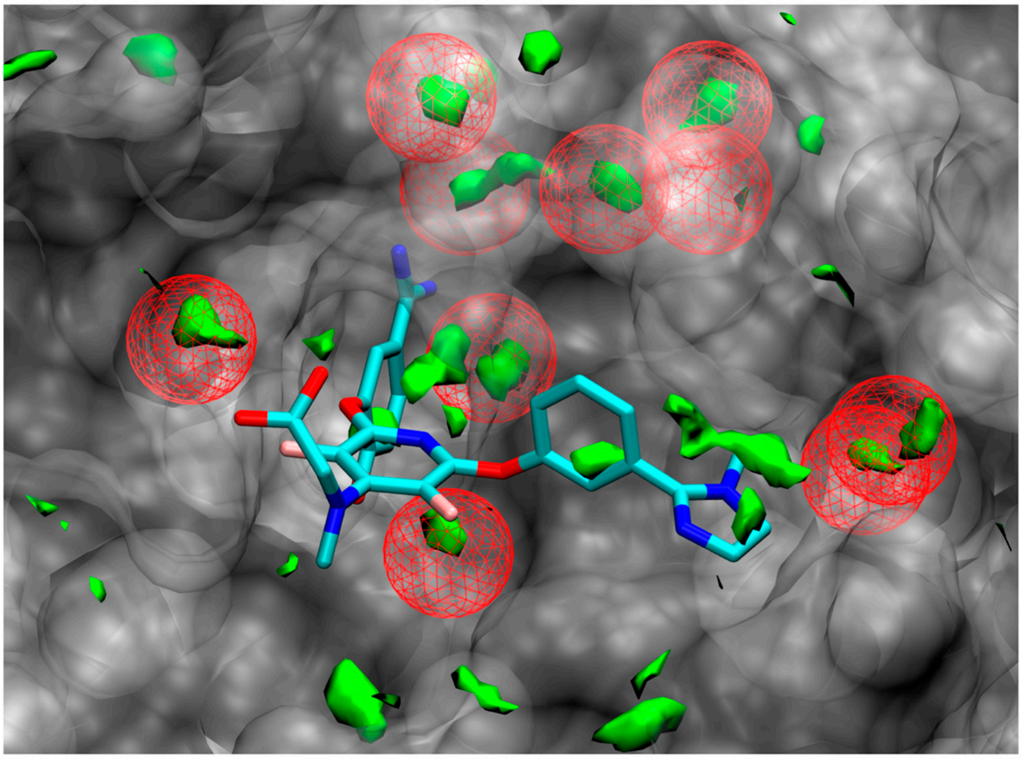

Figure S5. Co-crystal structure of water molecules (red sphere) in the active site of FXa (transparent surface) with a ligand (cyan stick) of PDB entry 1FJS. Green surface represents high-occupancy water region calculated by GIST.

References

1. Maignan, S.; Guilloteau, J.-P.; Choi-Sledeski, Y.M.; Becker, M.R.; Ewing, W.R.; Pauls, H.W.; Spada, A.P.; Mikol, V. Molecular structures of human factor Xa complexed with ketopiperazine inhibitors: Preference for a neutral group in the S1 pocket. *J. Med. Chem.* **2003**, *46*, 685–690.
2. Maignan, S.; Guilloteau, J.-P.; Pouzieux, S.; Choi-Sledeski, Y.M.; Becker, M.R.; Klein, S.I.; Ewing, W.R.; Pauls, H.W.; Spada, A.P.; Mikol, V. Crystal structures of human factor Xa complexed with potent inhibitors. *J. Med. Chem.* **2000**, *43*, 3226–3232.
3. Adler, M.; Davey, D.D.; Phillips, G.B.; Kim, S.H.; Jancarik, J.; Rumennik, G.; Light, D.R.; Whitlow, M. Preparation, characterization, and the crystal structure of the inhibitor ZK-807834 (CI-1031) complexed with factor Xa. *Biochemistry* **2000**, *39*, 12534–12542.
4. Nar, H.; Bauer, M.; Schmid, A.; Stassen, J.-M.; Wienen, W.; Priepke, H.W.; Kauffmann, I.K.; Ries, U.J.; Hauel, N.H. Structural basis for inhibition promiscuity of dual specific thrombin and factor Xa blood coagulation inhibitors. *Structure* **2001**, *9*, 29–37.
5. Guertin, K.R.; Gardner, C.J.; Klein, S.I.; Zulli, A.L.; Czekaj, M.; Gong, Y.; Spada, A.P.; Cheney, D.L.; Maignan, S.; Guilloteau, J.-P.; et al. Optimization of the β-Aminoester class of factor Xa inhibitors. Part 2: Identification of FXV673 as a potent and selective inhibitor with excellent In vivo anticoagulant activity. *Bioorg. Med. Chem. Lett.* **2002**, *12*, 1671–1674.
6. Matter, H.; Defossa, E.; Heinelt, U.; Blohm, P.-M.; Schneider, D.; Müller, A.; Herok, S.; Schreuder, H.; Liesum, A.; Brachvogel, V.; et al. Design and quantitative structure-activity relationship of 3-amidinobenzyl-1 h -indole-2-carboxamides as potent, nonchiral, and selective inhibitors of blood coagulation factor Xa. *J. Med. Chem.* **2002**, *45*, 2749–2769.
7. Ye, B.; Arnaiz, D.O.; Chou, Y.-L.; Griedel, B.D.; Karanjawala, R.; Lee, W.; Morrissey, M.M.; Sacchi, K.L.; Sakata, S.T.; Shaw, K.J.; et al. Thiophene-anthranilamides as highly potent and orally available factor Xa inhibitors 1. *J. Med. Chem.* **2007**, *50*, 2967–2980.
8. Quan, M.L.; Lam, P.Y.S.; Han, Q.; Pinto, D.J.P.; He, M.Y.; Li, R.; Ellis, C.D.; Clark, C.G.; Teleha, C.A.; Sun, J.-H.; et al. Discovery of 1-(3′-Aminobenzisoxazol-5′-yl)-3-trifluoromethyl-*N*-[2-fluoro-4-[(2′-dimethylaminomethyl) imidazol-1-yl]phenyl]-1H-pyrazole-5-carboxyamide Hydrochloride (Razaxaban), a highly potent, selective, and orally bioavailable factor Xa inhibitor. *J. Med. Chem.* **2005**, *48*, 1729–1744.

9. Matter, H.; Will, D.W.; Nazaré, M.; Schreuder, H.; Laux, V.; Wehner, V. Structural Requirements for factor Xa inhibition by 3-Oxybenzamides with neutral P1 substituents: Combining X-ray crystallography, 3D-QSAR, and tailored scoring functions. *J. Med. Chem.* **2005**, *48*, 3290–3312.
10. Nazaré, M.; Essrich, M.; Will, D.W.; Matter, H.; Ritter, K.; Urmann, M.; Bauer, A.; Schreuder, H.; Dudda, A.; Czech, J.; et al. Factor Xa inhibitors based on a 2-carboxyindole scaffold: SAR of neutral P1 substituents. *Bioorg. Med. Chem. Lett.* **2004**, *14*, 4191–4195.
11. Schärer, K.; Morgenthaler, M.; Paulini, R.; Obst-Sander, U.; Banner, D.W.; Schlatter, D.; Benz, J.; Stihle, M.; Diederich, F. Quantification of Cation-π interactions in protein-ligand complexes: Crystal-structure analysis of factor Xa bound to a quaternary ammonium ion ligand. *Angew. Chem. Int. Ed.* **2005**, *44*, 4400–4404.
12. Young, R.J.; Campbell, M.; Borthwick, A.D.; Brown, D.; Burns-Kurtis, C.L.; Chan, C.; Convery, M.A.; Crowe, M.C.; Dayal, S.; Diallo, H.; et al. Structure- and property-based design of factor Xa inhibitors: Pyrrolidin-2-ones with acyclic alanyl amides as P4 motifs. *Bioorg. Med. Chem. Lett.* **2006**, *16*, 5953–5957.
13. Pinto, D.J.P.; Galemmo, R.A.; Quan, M.L.; Orwat, M.J.; Clark, C.; Li, R.; Wells, B.; Woerner, F.; Alexander, R.S.; Rossi, K.A.; et al. Discovery of potent, efficacious, and orally bioavailable inhibitors of blood coagulation factor Xa with neutral P1 moieties. *Bioorg. Med. Chem. Lett.* **2006**, *16*, 5584–5589.
14. Senger, S.; Convery, M.A.; Chan, C.; Watson, N.S. Arylsulfonamides: A study of the relationship between activity and conformational preferences for a series of factor Xa inhibitors. *Bioorg. Med. Chem. Lett.* **2006**, *16*, 5731–5735.
15. Mueller, M.M.; Sperl, S.; Stürzebecher, J.; Bode, W.; Moroder, L. (*R*)-3-Amidinophenylalanine-Derived inhaibitors of Factor Xa with a novel active-site binding mode. *Biol. Chem.* **2002**, *383*, 1185–1191.
16. Kamata, K.; Kawamoto, H.; Honma, T.; Iwama, T.; Kim, S.H. Structural basis for chemical inhibition of human blood coagulation factor Xa. *Proc. Natl. Acad. Sci. USA* **1998**, *95*, 6630–6635.
17. Salonen, L.M.; Bucher, C.; Banner, D.W.; Haap, W.; Mary, J.-L.; Benz, J.; Kuster, O.; Seiler, P.; Schweizer, W.B.; Diederich, F. Cation-π interactions at the active site of factor Xa: Dramatic enhancement upon stepwise n-alkylation of ammonium ions. *Angew. Chem. Int. Ed.* **2009**, *48*, 811–814.
18. Pinto, D.J.P.; Orwat, M.J.; Koch, S.; Rossi, K.A.; Alexander, R.S.; Smallwood, A.; Wong, P.C.; Rendina, A.R.; Luettgen, J.M.; Knabb, R.M.; et al. Discovery of 1-(4-Methoxyphenyl)-7-oxo-6-(4-(2-oxopiperidin-1-yl)phenyl)-4,5,6,7-tetrahydro-1H-pyrazolo[3,4-c]pyridine-3-carboxamide (Apixaban, BMS-562247), a highly potent, selective, efficacious, and orally bioavailable inhibitor of blood coagulati. *J. Med. Chem.* **2007**, *50*, 5339–5356.
19. Young, R.J.; Borthwick, A.D.; Brown, D.; Burns-Kurtis, C.L.; Campbell, M.; Chan, C.; Charbaut, M.; Chung, C.; Convery, M.A.; Kelly, H.A.; et al. Structure and property based design of factor Xa inhibitors: Pyrrolidin-2-ones with biaryl P4 motifs. *Bioorg. Med. Chem. Lett.* **2008**, *18*, 23–27.
20. Zbinden, K.G.; Anselm, L.; Banner, D.W.; Benz, J.; Blasco, F.; Décoret, G.; Himber, J.; Kuhn, B.; Panday, N.; Ricklin, F. Design of novel aminopyrrolidine factor Xa inhibitors from a screening hit. *Eur. J. Med. Chem.* **2009**, *44*, 2787–2795.
21. Kleanthous, S.; Borthwick, A.D.; Brown, D.; Burns-Kurtis, C.L.; Campbell, M.; Chaudry, L.; Chan, C.; Clarte, M.-O.; Convery, M.A.; Harling, J.D.; et al. Structure and property based design of factor Xa inhibitors: pyrrolidin-2-ones with monoaryl P4 motifs. *Bioorg. Med. Chem. Lett.* **2010**, *20*, 618–622.
22. Salonen, L.M.; Holland, M.C.; Kaib, P.S.J.; Haap, W.; Benz, J.; Mary, J.-L.; Kuster, O.; Schweizer, W.B.; Banner, D.W.; Diederich, F. Molecular recognition at the active site of factor Xa: Cation-π interactions, stacking on planar peptide surfaces, and replacement of structural water. *Chem. A Eur. J.* **2012**, *18*, 213–222.
23. Watson, N.S.; Adams, C.; Belton, D.; Brown, D.; Burns-Kurtis, C.L.; Chaudry, L.; Chan, C.; Convery, M.A.; Davies, D.E.; Exall, A.M.; et al. The discovery of potent and long-acting oral factor Xa inhibitors with tetrahydroisoquinoline and benzazepine P4 motifs. *Bioorg. Med. Chem. Lett.* **2011**, *21*, 1588–1592.
24. Young, R.J.; Adams, C.; Blows, M.; Brown, D.; Burns-Kurtis, C.L.; Chan, C.; Chaudry, L.; Convery, M.A.; Davies, D.E.; Exall, A.M.; et al. Structure and property based design of factor Xa inhibitors: Pyrrolidin-2-ones with aminoindane and phenylpyrrolidine P4 motifs. *Bioorg. Med. Chem. Lett.* **2011**, *21*, 1582–1587.
25. Pruitt, J.R.; Pinto, D.J.P.; Galemmo, R.A.; Alexander, R.S.; Rossi, K.A.; Wells, B.L.; Drummond, S.; Bostrom, L.L.; Burdick, D.; Bruckner, R.; et al. Discovery of 1-(2-Aminomethylphenyl)-3-trifluoromethyl-*N*-[3-fluoro-2′-(aminosulfonyl)[1,1′-biphenyl)]-4-yl]-1H-pyrazole-5-carboxyamide (DPC602), a potent, selective, and orally bioavailable factor Xa inhibitor 1. *J. Med. Chem.* **2003**, *46*, 5298–5315.
